# Inner-sphere *vs.* outer-sphere reduction of uranyl supported by a redox-active, donor-expanded dipyrrin†
†Electronic supplementary information (ESI) available: General procedures, synthetic details, NMR spectra, EPR spectra, IR spectra, electronic absorption and emission spectra, voltammetric data, X-ray crystallographic data, computational details and optimised geometries. CCDC 1446511, 1446512 and 1507634. For ESI and crystallographic data in CIF or other electronic format see DOI: 10.1039/c6sc02912d



**DOI:** 10.1039/c6sc02912d

**Published:** 2016-10-28

**Authors:** James R. Pankhurst, Nicola L. Bell, Markus Zegke, Lucy N. Platts, Carlos Alvarez Lamfsus, Laurent Maron, Louise S. Natrajan, Stephen Sproules, Polly L. Arnold, Jason B. Love

**Affiliations:** a EaStCHEM School of Chemistry , The University of Edinburgh , Joseph Black Building, David Brewster Road , Edinburgh , EH9 3FJ , UK . Email: jason.love@ed.ac.uk ; Email: polly.arnold@ed.ac.uk; b LPCNO , INSA , Université de Toulouse , 135, avenue de Rangueil , 31077 Toulouse cedex 4 , France; c Centre for Radiochemisty Research , School of Chemistry , The University of Manchester , Oxford Road , Manchester , M13 9PL , UK; d WestCHEM School of Chemistry , University of Glasgow , Glasgow , G12 8QQ , UK

## Abstract

The uranyl(vi) complex UO_2_Cl(L) of the redox-active, acyclic diimino-dipyrrin anion, L^–^ is reported and its reaction with inner- and outer-sphere reductants studied. Voltammetric, EPR-spectroscopic and X-ray crystallographic studies show that chemical reduction by the outer-sphere reagent CoCp_2_ initially reduces the ligand to a dipyrrin radical, and imply that a second equivalent of CoCp_2_ reduces the U(vi) centre to form U(v). Cyclic voltammetry indicates that further outer-sphere reduction to form the putative U(iv) trianion only occurs at strongly cathodic potentials. The initial reduction of the dipyrrin ligand is supported by emission spectra, X-ray crystallography, and DFT; the latter also shows that these outer-sphere reactions are exergonic and proceed through sequential, one-electron steps. Reduction by the inner-sphere reductant [TiCp_2_Cl]_2_ is also likely to result in ligand reduction in the first instance but, in contrast to the outer-sphere case, reduction of the uranium centre becomes much more favoured, allowing the formation of a crystallographically characterised, doubly-titanated U(iv) complex. In the case of inner-sphere reduction only, ligand-to-metal electron-transfer is thermodynamically driven by coordination of Lewis-acidic Ti(iv) to the uranyl oxo, and is energetically preferable over the disproportionation of U(v). Overall, the involvement of the redox-active dipyrrin ligand in the reduction chemistry of UO_2_Cl(L) is inherent to both inner- and outer-sphere reduction mechanisms, providing a new route to accessing a variety of U(vi), U(v), and U(iv) complexes.

## Abstract 2

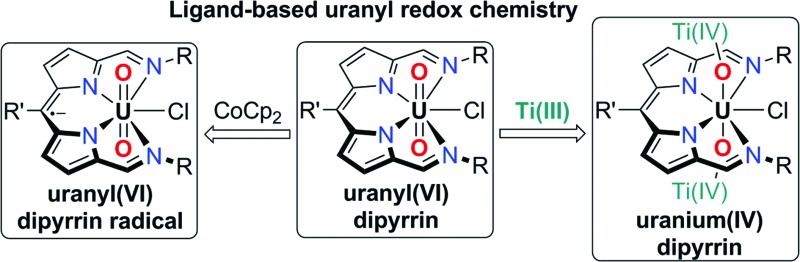
The uranyl(vi) complex UO_2_Cl(L) of the redox-active diimino-dipyrrin anion, L^–^ is reported and its reactions with inner- and outer-sphere reductants studied.

## Introduction

Redox-active ligands in metal complexes can provide alternative pathways in redox chemistry that are not available to metal complexes of more traditional, “innocent” ancillary ligands.^1–5^ While these features have been studied extensively in complexes of the transition metals, the use of redox-active ligands in actinide chemistry is less developed. Low oxidation-state uranium complexes, *i.e.* U(iii) and U(iv), of classical redox-active ligands such as pyridine di-imines (PDI), bipyridines, amidophenolates, and α-di-imines dominate and display reactivity in which ligand-centred redox processes are implicit.^6–15^ In contrast, compounds of higher oxidation state uranium *e.g.* uranyl(vi)/(v) with redox-active ligands are rare. This is surprising, as the reduction of uranyl(vi) to U(iv) is an important aspect of uranium remediation by immobilisation, and significant advances have been made in the reduction chemistry of uranyl(vi), *e.g.* forming oxo-metalated and oxo-silylated uranyl(v) compounds.^16–30^ Uranyl(vi) complexes of expanded porphyrins and analogous π-conjugated macrocycles are known but their impact in reduction chemistry has not been studied.^31,32^ The uranyl(vi) complex of an α-di-imine diphenolate undergoes single-electron reduction leading to the uranyl(vi) ligand-centred radical anion and not the expected uranyl(v) complex.^33^ Uranyl complexes of maleonitrile-containing Schiff-base complexes exhibit ligand-centred oxidation.^34^ Oxidation of a U^IV^Cp*(PDI) complex forms a uranyl(vi) complex of a PDI-ligand-centred radical anion which subsequently can undergo stoichiometric, stepwise reductive silylation by Me_3_SiI in which electrons arising from Cp* (through elimination of [Cp*]_2_) and the PDI ligand radical anion are involved.^9^

Molecular systems that favour an overall two-electron reduction of U(vi) to U(iv) are particularly sought after as U(iv) salts are generally less soluble than U(vi) and may provide a deeper understanding of uranium (bio)remediation. The reduction of uranyl(vi) β-ketoiminate complexes to U(iv) is facilitated by oxo-coordination of boron Lewis acids,^30^ and reductive silylation of a uranyl(vi) β-diketonate leads to the isolation of a U(iv) triflate through O-abstraction.^35^ Two-electron reduction of uranyl(vi) is thought to occur on photolysis of a phosphine oxide complex in the presence of alcohols, forming U(iv) alkoxide complexes,^36,37^ and comproportionation of U(vi) and U(iii) triflates has been shown to form U(iv) polyoxo clusters.^38^ Additionally, the reaction of simple Lewis bases with functionalised U(v) iodo complexes has led to further reduction to U(iv) on elimination of I_2_.^23^ Furthermore, bacterial species such as *Geobacter sulfurreducens* have demonstrated biotic reduction of [UO_2_]^2+^ to UO_2_ (uraninite);^39–41^ in comparison the [NpO_2_]^+^ congener is not reduced under these conditions.^42^

**Scheme 1:**
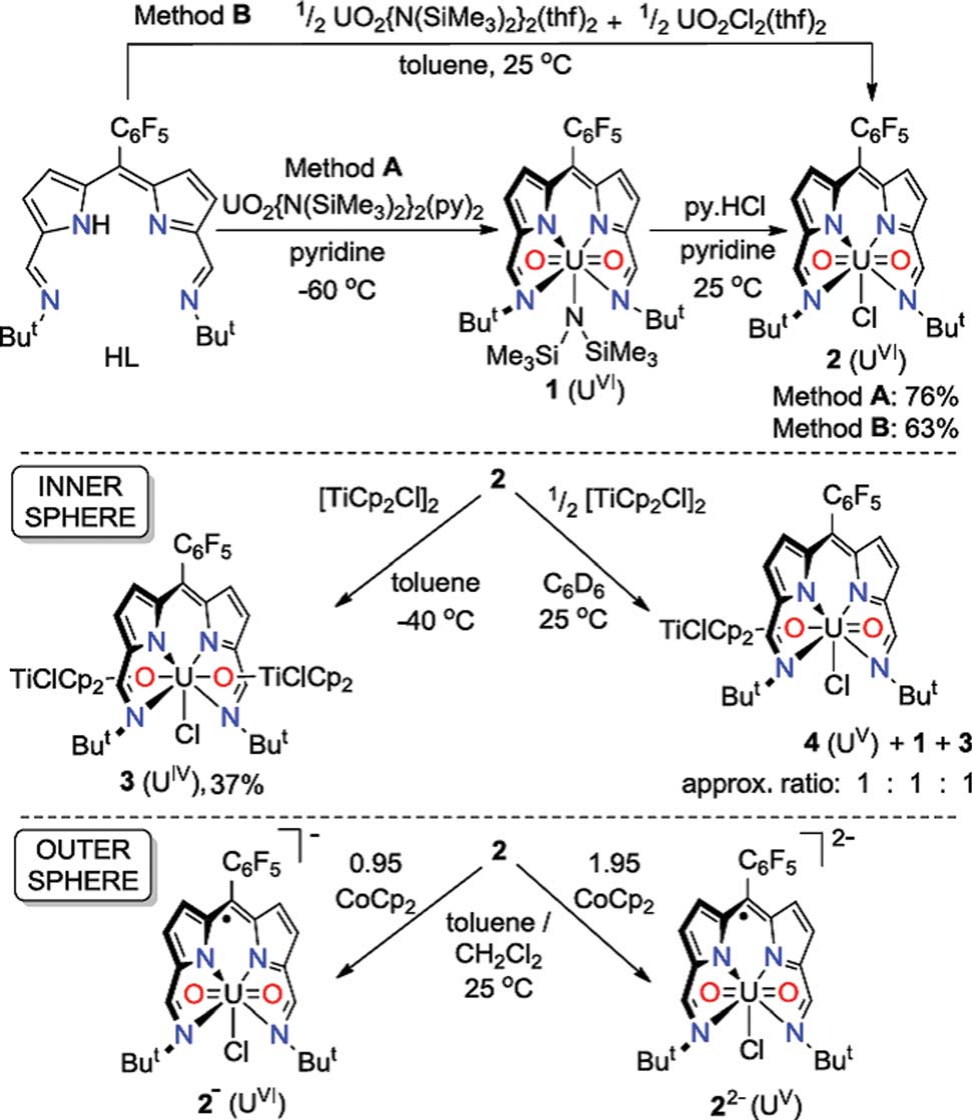
Synthesis of the uranyl(vi) complexes **1** and **2** and the U(iv) complex **3** (isolated yields shown) and reactions between **2** and the inner-sphere reductant, [TiCp_2_Cl]_2_ and the outer-sphere reductant, CoCp_2_.

We recently reported the straightforward synthesis of the mono-anionic, tetradentate dipyrrin ligand, HL (Scheme 1), and the solid-state structure of a related iron(iii) complex {FeBr(L)}_2_(μ-O) in which the iron centre is five-coordinate with one imine arm pendant.^43^ As such, we anticipated that L^–^ would occupy four of the five equatorial sites of the larger uranyl dication and that the incorporation of the potentially redox-active, donor-expanded dipyrrin framework would allow access to new, ligand-mediated, uranyl reduction chemistry. Here we report a new donor-expanded dipyrrin uranyl(vi) complex and its contrasting inner- and outer-sphere redox chemistry, both of which routes involve the redox-active dipyrrin.

## Results

### Synthesis and structures of uranyl(vi) complexes

**Fig. 1:**
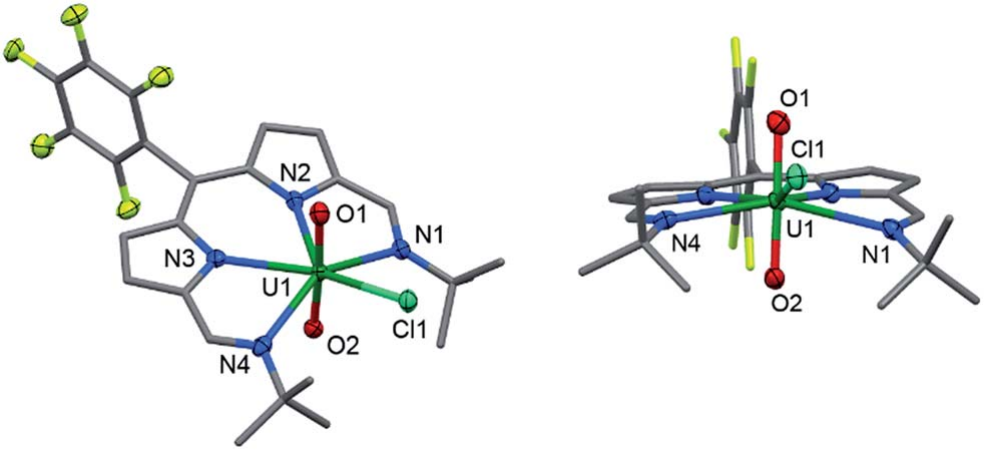
Solid-state structure of **2**. For clarity, all hydrogen atoms and two benzene solvent molecules are omitted (displacement ellipsoids drawn at 50% probability and fluorine atoms drawn in yellow). Selected distances/Å: U1–O1, 1.766(4); U1–O2, 1.763(4); U1–N1, 2.684(5); U1–N2, 2.465(5); U1–N3, 2.483(4); U1–N4, 2.681(5); U1–Cl1, 2.710(1). Selected angles/deg.: O1–U1–O2, 175.5(2); sum of equatorial bond angles around U1, 368.26.

The uranyl silylamide dipyrrin complex **1** was prepared *in situ* by the transamination reaction of HL with an equimolar amount of UO_2_{N(SiMe_3_)_2_}_2_(py)_2_ (Scheme 1, Method A). Compound **1** was not isolated, but instead reacted further with pyridinium chloride to give the uranyl chloride complex, **2**, which was isolated as a blue, highly moisture-sensitive solid in high overall yield (76%). Alternatively, reaction of HL with a 1 : 1 mixture of UO_2_{N(SiMe_3_)_2_}_2_(thf)_2_ and UO_2_Cl_2_(thf)_2_ in toluene yields **2** directly in high yield and purity (Method B). The ^1^H NMR spectrum of **2** in C_6_D_6_ features four resonances for the dipyrrin ligand, with the imine protons seen at 9.52 ppm, equivalent pyrrole protons as doublets at 7.30 ppm and 7.18 ppm and *tert*-butyl protons as a single resonance at 2.01 ppm. In the batch crystallised from method A three pyridine resonances, most likely from solvent of crystallisation are seen at 8.56, 7.65 and 7.25 ppm, integrating with a 1 : 1 ratio with **2**. When **2** is synthesised in the absence of pyridine (Method B) the NMR spectrum of crystalline material shows no solvent inclusion. In the ^19^F{^1^H} NMR spectrum, there is a single set of sharp resonances for the C_6_F_5_ *meso*-substituent, at –140.68, –155.19 and –163.34 ppm. Noteworthy absorption bands in the IR spectrum of **2** are those ascribed to the imine functional group at 1556 cm^–1^ and also the asymmetric stretching mode of the uranyl group (*ν*_3_) at 878 cm^–1^. Purple/blue, pleochroic single crystals of **2** grown from a concentrated benzene solution were suitable for structural analysis by X-ray crystallography (Fig. 1). In the solid state, **2** adopts a distorted pentagonal bipyramidal coordination geometry, in which the N_4_-donor set of the donor-expanded dipyrrin ligand occupies the equatorial positions along with the chloride ligand, with the equatorial bond angles summing to 368.26°. The Cl1 centre is situated 1.647 Å above the mean N_4_-plane. This distortion from its position in the idealised geometry means that steric interactions between the chloride ligand and the nearby *tert*-butyl substituents are minimised. These *tert*-butyl groups bend away from the same face of the N_4_ donor-plane, meaning that the *C*_2v_ symmetry observed in solution is not retained in the solid state. At 1.766(4) and 1.763(4) Å, the two U–O bond distances are identical and are typical for a U(vi) uranyl complex, and the O–U–O bond angle is essentially linear at 175.5(2)°.

### Synthesis and structure of the U(iv) complex

**Fig. 2:**
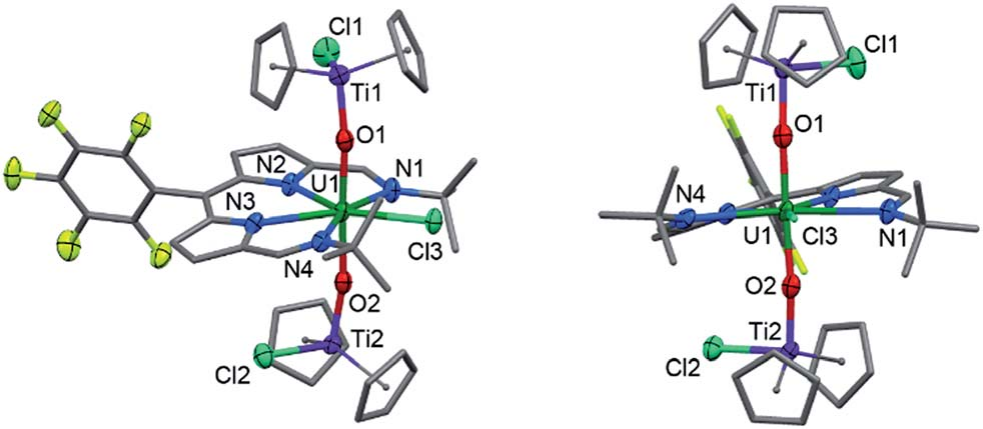
Solid-state structure of **3**. For clarity, all hydrogen atoms and one benzene solvent molecule are omitted (displacement ellipsoids drawn at 50% probability and fluorine atoms drawn in yellow). Selected distances/Å: U1–O1, 2.066(7); U1–O2, 2.062(7); U1–N1, 2.806(7); U1–N2, 2.530(6); U1–N3, 2.531(7); U1–N4, 2.815(6); U1–Cl1, 2.713(2); Ti–O, 1.841(7); mean Ti–Cp centroid, 2.08; Ti–Cl, 2.373(3). Selected angles/deg.: O1–U1–O2, 177.0(2); sum of equatorial bond angles around U, 360.57.

Reaction of **2** with one equivalent of [TiCp_2_Cl]_2_ (Scheme 1) results in the Ti-oxo-functionalised U(iv) complex [(TiCp_2_Cl)–OUO–(TiCp_2_Cl)(Cl)(L)] **3**, a blue compound which exhibits paramagnetically-shifted ^1^H NMR resonances in *d*_8_-THF from –40 ppm to +50 ppm. A single *tert*-butyl resonance appears at –31.68 ppm with an imine singlet at –37.33 ppm. Resonances at –17.90 and –22.80 ppm are assigned to pyrrole protons based on magnetisation transfer observed by correlation spectroscopy. A single ^1^H NMR resonance at 43.63 ppm, integrating to 20H, is consistent with four cyclopentadienyl (Cp) groups per L. A single set of resonances in the ^19^F{^1^H} NMR spectrum are observed at –153.48, –161.97 and –170.44 ppm. Blue crystals of **3** were grown from a benzene solution and the solid-state structure was determined by X-ray crystallography (Fig. 2).

In the solid-state **3** is clearly a neutral molecule, therefore the uranium and titanium centres are both assigned formal oxidation states of +4. The uranium centre in **3** adopts a more regular pentagonal bipyramidal coordination geometry than that found in **2**, with the equatorial bond angles summing to 360.6°. This may be due to the *tert*-butyl substituents adopting a *C*_2_-twist in **3**. The U–N bond distances in **3** are all elongated by 0.06 to 0.12 Å compared to those in **2**, consistent with reduction of U(vi) to a lower oxidation state. Importantly, the U–O bond lengths (2.066(7)–2.062(7) Å) are elongated significantly compared to those in **2** and are similar to U(iv) siloxy complexes (2.065 Å)^23^ but longer than U(v) siloxy complexes (1.993 Å).^19^ Single U–O bonds are on average 2.361 Å (CCD search with 3028 relevant complexes) which suggests that the U–O bonds in **3** retain some multiple-bond character. The Ti1–O1 bond distance of 1.841(7) Å is similar to that found in the Ti(iv) oxo-bridged dimer [TiCp_2_Cl]_2_(μ-O) (1.837(2) Å).^44^

### Attempted synthesis of uranyl(v) complexes

Reactions between **2** and 0.5 equivalents of [TiCp_2_Cl]_2_ in C_6_D_6_ or *d*_8_-THF did not lead to clean conversion to the uranyl(v) complex [(TiCp_2_Cl)–OUO(Cl)(L)] **4** (Scheme 1) but instead led to a mixture of complexes, comprising **4**, the starting material **2**, and the U(iv) complex **3** in *ca.* a 1 : 1 : 1 ratio, with the latter complex precipitating from solution over time. The ratios of complexes in this mixture change slowly over 24 h in solution to generate more of **2** and **3** (the latter as a precipitate), *i.e.* **4** undergoes slow disproportionation. The uranyl(v) complex is identified by its ^1^H NMR spectrum only, with characteristic resonances between +20 and –10 ppm, in particular the singlet resonances at 19.35 ppm for 10 Cp protons and –8.58 ppm for 18 ^*t*^Bu protons.

### Electrochemistry

**Fig. 3:**
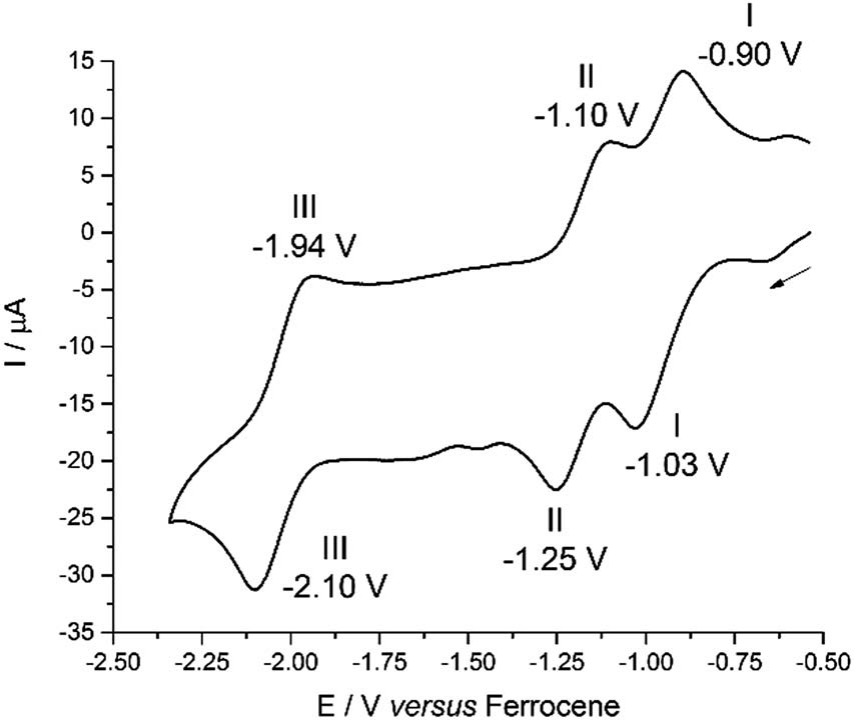
Cyclic voltammogram for **2** measured as a 1 mM CH_2_Cl_2_ solution (0.1 M [^*n*^Bu_4_N][BPh_4_] supporting electrolyte, glassy-carbon working electrode, Pt gauze counter electrode and silver wire quasi-reference electrode). Potentials are referenced against the Fc^+^/Fc couple measured under identical conditions.

The cyclic voltammogram (CV) of **2** was measured in CH_2_Cl_2_ and, at a scan-rate of 100 mV s^–1^, features three quasi-reversible reduction processes at *E*_1/2_ –0.97, –1.18 and –2.02 V *vs.* Fc^+^/Fc (Fig. 3). For all three reductions, corresponding oxidation waves were observed with peak-to-peak separations of 150 mV. Processes I and II are separated by 210 mV and process III occurs close to the cathodic edge of the electrochemical window rendering comparison of the peak areas unreliable. In the square-wave voltammogram (see ESI†), the three reduction processes were measured at *E*_1/2_ –0.96, –1.17 and –2.02 V *vs.* Fc^+^/Fc, and the return oxidation waves were measured at identical potentials. For all three reduction processes, 92–98% of the charge passed in the forward cathodic scan was passed back in the return anodic scan.

For comparison, the CV of HL and the potassium salt, KL, were measured under identical conditions (see ESI†). HL undergoes two irreversible reduction processes at *E*cp –1.50 V and –2.07 V *vs.* Fc^+^/Fc and KL also undergoes two irreversible reduction processes, at *E*cp –1.29 V and –1.57 V.

### EPR spectroscopy

Although CoCp_2_ is a strong outer-sphere reductant with a formal Co(iii)/Co(ii) redox potential of –1.33 V *vs.* Fc^+^/Fc,^48^ and could be used to carry out both reduction processes I and II (Fig. 3), the first reduction can be achieved selectively using a sub-stoichiometric quantity of reductant. The reaction between **2** and 0.95 equivalents of CoCp_2_ forms a NMR-silent, magenta solution of **2^–^** in C_6_D_6_. The EPR spectrum of **2^–^** shows a relatively sharp line devoid of hyperfine structure synonymous with the formation of an *S* = 1/2 species (Fig. S31 and S32†). The spectral profile was not improved by cooling the sample to 223 K. Simulation yielded *g*_iso_ 1.9893, a value significantly shifted from that of the free electron (2.0023). This spectrum is consistent with a ligand-centred reduction affording [U^VI^O_2_Cl(L˙)]^–^, where the presence of the attached U(vi) ion not only instigates the *g*-shift but broadens the line, obscuring all hyperfine splitting from the various spin-active nuclei in the dipyrrin. Similar shifts in *g*-value and line broadening have been reported previously for fluid solution spectra of U(vi)-(L˙) species.^9,49^ Moreover, the observation of a signal at ambient temperature is incongruous with the formation of U(v) whose 5f^1^ configuration displays drastically different magnetic properties.^50^ The frozen solution spectrum collected at 130 K is highly isotropic as evinced by *g* = (1.9974, 1.9872, 1.9786) obtained from the simulation (see ESI†). The lack of *g*-anisotropy and relatively modest *g*-shift attests to the limited U character in the LUMO of **2** (see below). Furthermore, *in situ* reduction of **2** with 1 equiv. [TiCp_2_Cl]_2_ (*i.e.* 2 × Ti(iii)) did not exhibit any EPR (nor diamagnetic NMR) signals, including any from trace Ti(iii) reductant. This supports the Ti(iv)–U(iv)–Ti(iv) electronic structure assignment of **3**.

### Electronic spectroscopy

**Fig. 4:**
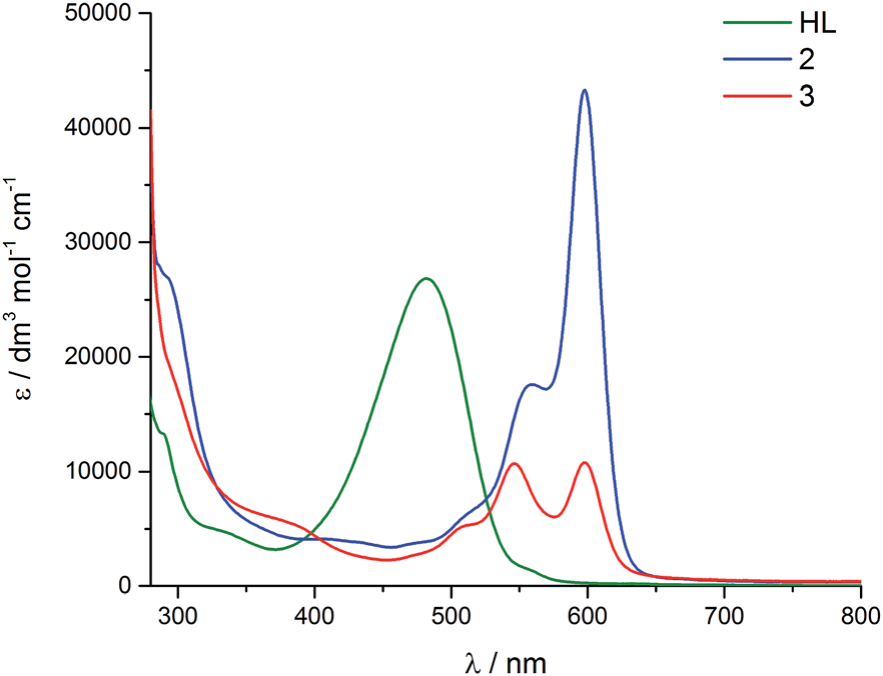
Room-temperature electronic absorption spectra for HL (green), **2** (blue) and **3** (red), all measured as toluene solutions.

**Fig. 5:**
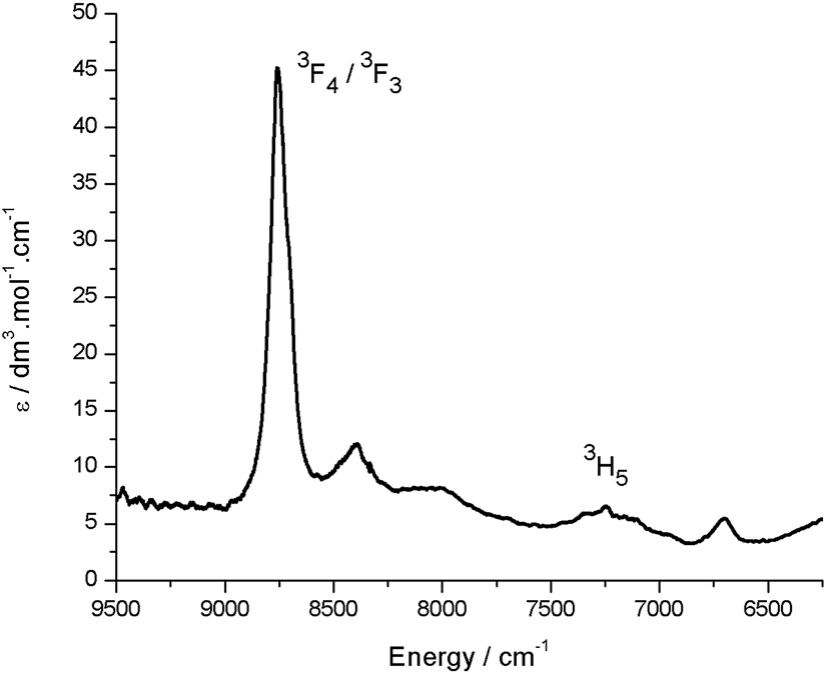
Room temperature NIR absorption spectrum for **3** (toluene solution). Assignments are based on comparison with U(iv) pentahalide anions, which display near-identical NIR spectra.^45^

In the visible region, the lowest-energy absorption band for HL appears with *λ*_max_ at 485 nm (Fig. 4).^43^ Upon coordination to the uranyl group in **2**, this lowest-energy absorption is bathochromically (red) shifted, with two bands observed at 598 nm and 557 nm, and a shoulder at 511 nm. The absorption band at 598 nm represents *ε*_max_ and has a value of 43 000 dm^3^ mol^–1^ cm^–1^. The reduced uranium(iv) complex **3** has a near-identical absorption spectrum to **2**, albeit with a significantly reduced molar absorptivity (*ε*_max_ = 11 000 dm^3^ mol^–1^ cm^–1^ at 598 nm). Complex **3** also features a number of weak absorption bands in the near-infra-red (NIR) region with extinction coefficients less than 50 dm^3^ mol^–1^ cm^–1^ (Fig. 5). This pattern is nearly identical to U(iv) pentahalide anions^45,46^ and supports the +4 oxidation state for complex **3**. The higher-energy f–f transitions for the ground-state ^3^H_4_ ion are not observed due to the intense absorption of the dipyrrin chromophore in the UV and visible regions.

**Fig. 6:**
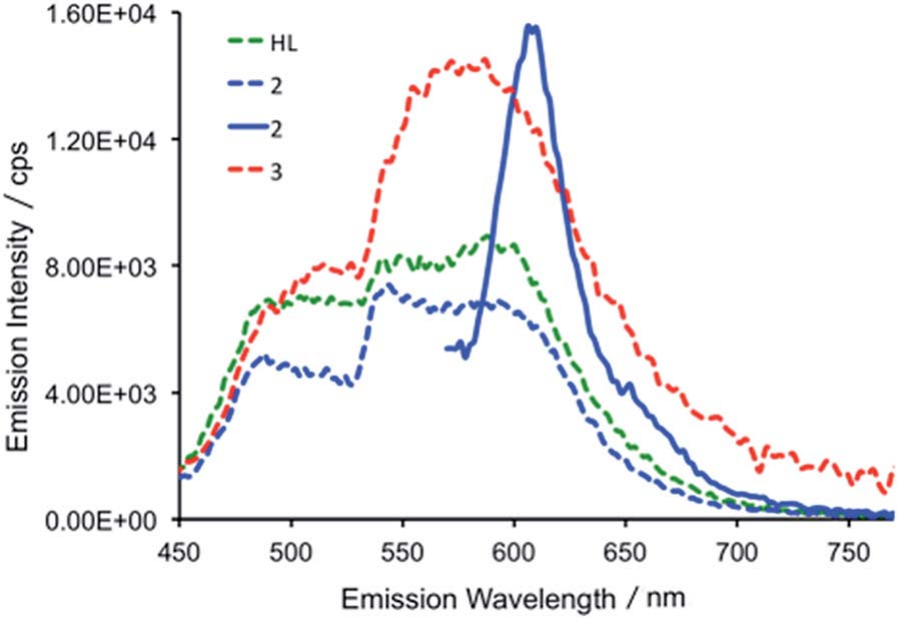
Emission spectra of HL (green dashed trace), **2** (blue dashed trace) and **3** (red dashed trace) following excitation at 425 nm and the emission spectrum of **2** (full blue line) following 550 nm excitation (298 K, THF). The relative intensities of HL, **2** and **3** are arbitrary with respect to each other.

At room temperature, excitation into the dipyrrin absorption bands in HL dissolved in THF at 280, 405, 425 and 550 nm results in fluorescence spectra with emission bands centred at 490 and 600 nm in all cases (Fig. 6).

The fluorescence lifetimes of the emission features for HL were recorded following 405 nm excitation with a picosecond pulsed diode LASER and were measured in the nanosecond regime (see Table S2†).^47^ All lifetimes fit well to biexponential decay kinetics, with components of *ca.* 1 and 5 ns. In a frozen glass at 77 K, following excitation at 240, 405 and 550 nm, the resulting emission spectra are essentially identical to those recorded in fluid solution at room temperature. The fluorescence lifetimes at 77 K are similar to those recorded in fluid solution and again are biexponential at *ca.* 1 and 4 ns for both the emission at 490 and 600 nm.

In the uranium complexes **2** and **3**, excitation into the dipyrrin ligand absorption bands at 240, 405 and 425 nm affords spectra that are similar to those of HL (Fig. 6). However, for complex **2**, the larger molar absorption coefficient in the band at *ca.* 593 nm enabled us to spectrally isolate the emission arising from this transition. The recorded emission spectrum is essentially a mirror image of the absorption band with only a marginal Stokes shift (*ca.* 8 nm). Room temperature time-resolved measurements enabled the luminescence lifetimes of all the emission bands in **2** and **3** to be measured upon excitation at 405 nm, and these are found to be biexponential and comparable to those measured for HL in fluid solution (see Table S2†). Unfortunately, accurate lifetime data were not obtained for complexes **2** and **3** at 77 K due to the comparatively weaker emission intensities compared to HL in frozen THF in optically dilute samples.

**Fig. 7:**
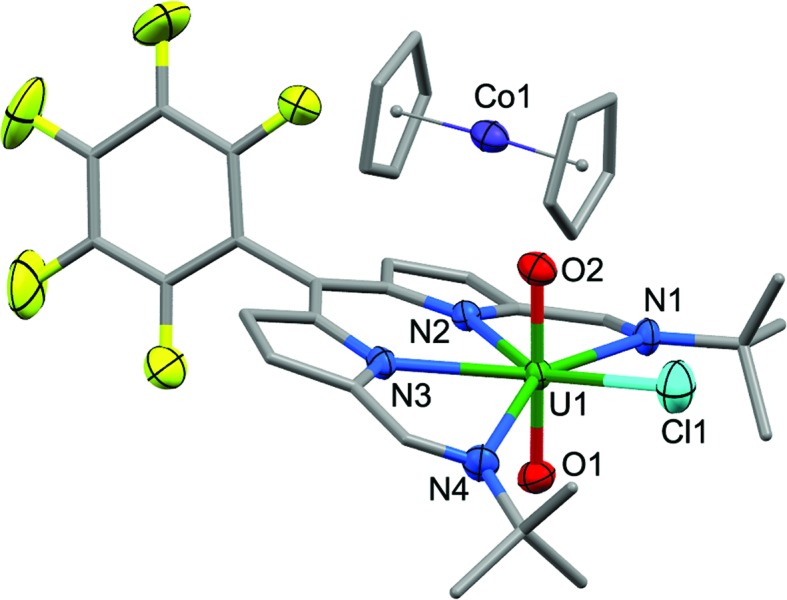
Solid-state structure of the singly reduced [CoCp_2_][**2**]. For clarity, all hydrogen atoms, one THF solvent molecule, and a second molecule of [CoCp_2_][**2**] from the asymmetric unit are omitted (displacement ellipsoids drawn at 50% probability and fluorine atoms drawn in yellow). Selected distances/Å: U1–O1, 1.772(3); U1–O2, 1.779(3); U1–N1, 2.689(4); U1–N2, 2.482(3); U1–N3, 2.461(3); U1–N4, 2.670(4); U1–Cl1, 2.648(1). Selected angles/deg.: O1–U1–O2, 176.5(1); sum of equatorial bond angles around U, 364.9.

The *in situ* reduction of **2** by one and two equivalents of CoCp_2_ in toluene was carried out and analysed by UV-vis-NIR spectrophotometry. Reaction of **2** with one equivalent of CoCp_2_ caused a significant hypsochromic (blue) shift in the band at 557 nm (*ε* = 43 000 dm^3^ mol^–1^ cm^–1^) in **2** to 548 nm (27 000) in **2^–^**. Addition of a second equivalent of CoCp_2_ forming **2^2–^** caused a further hypsochromic shift to 539 nm (26 000). A significant decrease in the extinction coefficient of these features occur on reduction of **2** to **2^–^**. While attempts to isolate and crystallise **2^2–^** were unsuccessful, a small quantity of crystals were isolated from the reaction between **2** and 2 CoCp_2_ and proved to be the singly reduced species **2^–^** (Fig. 7). The UV-vis spectrum of this crystalline material is identical to that resulting from the *in situ* reaction between **2** and one equivalent of CoCp_2_ and is very different to the spectra for **2** and **2^2–^** (see ESI†).

### Structure of [CoCp_2_][**2**]

The X-ray crystal structure of [CoCp_2_][**2**] shows one cobaltocenium cation per uranyl dipyrrin complex, indicative of single-electron reduction, with two near identical molecules in the asymmetric unit, therefore only one is considered. The uranyl oxo bond distances U1–O1 and U1–O2 are 1.772(3) and 1.779(3) Å, respectively, and it is clear from these bond distances that reduction of the metal has not occurred. Instead, some changes are seen in the bonding within the dipyrrin structure although these could be viewed as statistically insignificant to 3*σ*. In general, the changes in bonding match those expected from the LUMO of **2** (see below), in which there is a shortening of the bonds for bonding components in the LUMO whereas anti-bonding components result in bond elongation, as expected for the addition of electron density into the LUMO.

### DFT calculations

**Fig. 8:**
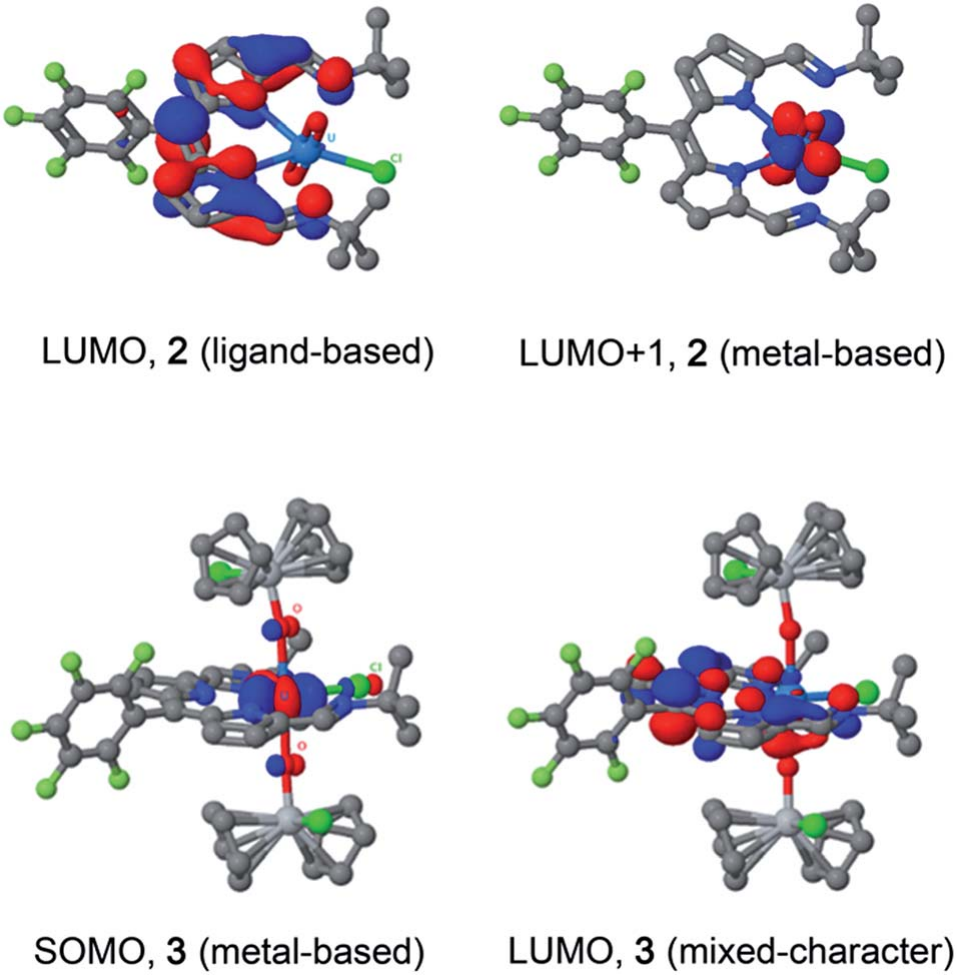
LUMO and LUMO+1 of U(vi) complex **2** (top), and SOMO and LUMO of U(iv) complex **3** (bottom).

Molecular orbital calculations reveal that the LUMO of **2** is located entirely on the ligand (Fig. 8) whereas the LUMO+1 is metal based (higher in energy by 19.7 kcal mol^–1^ with respect to the LUMO). The first inner-sphere reduction of **2** can occur in two ways: either a direct reduction of the uranium centre to yield the final (L)–U(v)–Ti(iv) complex or in a two-step fashion with the formation of a (L˙)–U(vi)–Ti(iv) intermediate that ultimately forms the (L)–U(v)–Ti(iv) compound. A second inner-sphere reduction of the (L)–U(v)–Ti(iv) compound would result in the formation of the observed (L)–U(iv)–Ti(iv)_2_ product **3**.

Since the LUMO of complex **2** is located solely on the ligand, a ligand-based reduction was first investigated computationally by optimising the geometry of the proposed (L˙)–U(vi)–Ti(iv) intermediate. The nature of the oxidation state of the uranium centre was fixed at +6 using f-in-core Relativistic Effective Core Potentials (RECP). Such an intermediate was computed to be stable and its formation is exergonic by 5.7 kcal mol^–1^ with respect to the separated reactants (**2** + TiCp_2_Cl). Relaxing the oxidation state of the uranium centre by performing a small-core calculation, *i.e.* without imposing the f-configuration, leads to the formation of a more stable (L)–U(v)–Ti(iv) complex that is 4.0 kcal mol^–1^ lower in energy than the (L˙)–U(vi)–Ti(iv) intermediate. The first reduction is therefore occurring in two steps with first a ligand-based reduction that evolves into a metal-based reduction with the formation of a (L)–U(v)–Ti(iv) complex. For the second reduction and the formation of complex **3**, the situation is slightly different as the LUMO of the (L)–U(v)–Ti(iv) intermediate exhibits an overlap between f-orbitals of U and the π*-orbital of the ligand. Therefore, estimating the difference between ligand-based and metal-based reduction is not straightforward computationally. Even so, the second reduction is found to be exergonic by 12.5 kcal mol^–1^ with respect to (L)–U(v)–Ti(iv). Furthermore, the optimised structure of **3** is in close agreement with that determined crystallographically.

An alternative disproportionation of the (L)–U(v)–Ti(iv) intermediate to generate a 1 : 1 mixture of **2** and **3** was also evaluated and was found to be 11.2 kcal mol^–1^ higher in energy than sequential reduction by Ti(iii). Finally, the plausibility of an outer-sphere reduction mechanism was investigated. The first step would be a ligand-based reduction yielding [(L˙)–U(vi)]^–^, followed by two metal-based reductions yielding a [(L˙)–U(iv)]^3–^ complex through the formation of a [(L˙)–U(v)]^2–^ compound. The ligand-based reduction with respect to CoCp_2_ is computed to be favourable by 37.1 kcal mol^–1^, and the two metal-based reductions are exergonic by 56.4 kcal mol^–1^ and 57.0 kcal mol^–1^ respectively.

## Discussion

The reductive functionalisation of the uranyl complex **2** by two chloro-titanocene units to afford the U(iv) complex **3** is supported by the presence of four Cp ligands per dipyrrin in the NMR spectra of **3**. While the mono-titanated uranyl(v) complex **4** is identified along with **2** and **3** in the ^1^H NMR spectrum using sub-stoichiometric Ti(iii) reductant, it could not be isolated and undergoes very slow disproportionation; in contrast, the formation of **3** is rapid. The reduction of **2** to form U(iv) (**3**) is supported by the absorption band at 630 cm^–1^ in the IR spectrum of **3**, assigned to the asymmetric stretching mode of the neutral UO_2_ moiety,^23,26^ which is 250 cm^–1^ lower in energy than that of **2**, but *ca.* 200 cm^–1^ higher in energy than the equatorial U–O single bonds in U(vi)–alkoxide and –thiocarbamato complexes.^51^ Furthermore, the solid-state structure of **3** is consistent with that of a U(iv) complex ligated by a singlet dipyrrin ligand. An overlay of the crystal structures reveals that the dipyrrin ligand in **3** is structurally identical to that in **2** (see ESI†), indicating that **3** is not a U(v) complex of a dipyrrin ligated by a dipyrrin radical.

The presence of the dipyrrin chromophore in HL, **2** and **3** dominates the electronic spectra of these compounds and possible uranium emission,^52^ and impacts on their redox processes. For **2**, the band at 557 nm is attributed to a charge-transfer (CT) transition due to its small hypsochromic shift on increasing solvent polarity. Solvatochromism was not observed for the band at 598 nm and this band is therefore assigned to a dipyrrin-based π–π* transition, a common feature for dipyrrin compounds and their transition metal complexes.^53–55^ In support of this latter assignment, DFT calculations show that both the HOMO and LUMO in **2** are ligand-based. A group theoretical analysis of the frontier set of molecular orbitals for **2**, assuming *C*_2v_ symmetry in solution as observed in the ^1^H NMR spectrum, reveals that many of the transitions departing from the occupied frontier orbitals are orbitally allowed. This includes the HOMO–LUMO transition and gives rise to an intense absorption with *ε*_max_ of 42 000 dm^3^ mol^–1^ cm^–1^.

The involvement of ligand redox processes in the reduction of **2** is supported by cyclic voltammetry, EPR, DFT and X-ray crystallographic measurements. The uranyl complex **2** undergoes a total of three quasi-reversible reduction processes, the first two of which are separated by 210 mV and both lie in the region of previously reported U(vi)/U(v) uranyl redox couples.^20,23,56,57^ The observation of an organic radical with a simulated *g*_iso_ value of 1.9893 on chemical reduction of **2** with CoCp_2_ confirms that the first outer-sphere reduction, observed by CV to occur at *E*_1/2_ –0.97 V *vs.* Fc^+^/Fc, is due to the formation of the dipyrrin-radical U(vi) complex **2^–^**. The lack of *g*-anisotropy and relatively small *g*-shift attests to the essentially zero U-character in the LUMO of **2** from the DFT calculations. This conclusion is also supported by the solid-state structure of **2^–^** which displays no elongation of the uranyl oxo bonds expected for metal-centred reduction but some variation in dipyrrin bonding consistent with ligand-based reduction (see ESI†). The second outer-sphere reduction, observed at *E*_1/2_ –1.18 V, is therefore best assigned to the formation of a dipyrrin-radical U(v) complex. Monitoring the UV-vis spectra of the *in situ* reduction of **2** with one and two equivalents of CoCp_2_ supports these assignments, as distinct hypsochromic shifts and a decrease in the extinction coefficients of the charge transfer band are observed.^58,59^ No NIR absorptions are seen on reduction with one equivalent of CoCp_2_, supporting a ligand-based reduction in that case. NIR absorptions were also not observed following reaction of **2** with two equivalents of CoCp_2_; formation of U(v) should give rise to f–f transitions in the NIR region in this case. However, the product of this two-electron reduction is paramagnetic and NMR-silent, and so this second reduction cannot also be ligand-based, as such a reduction would form a diamagnetic, closed-shell ligand dianion coordinated to U(vi).

**Scheme 2:**
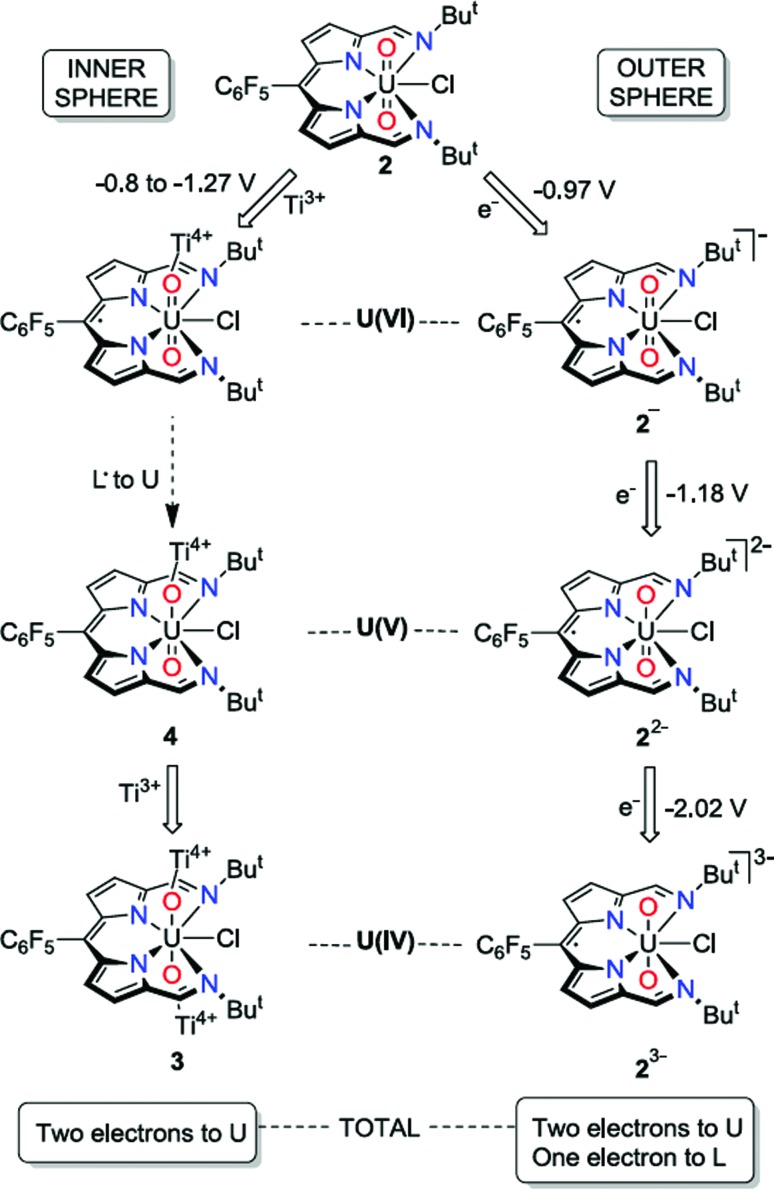
Contrasting outer- and inner-sphere reduction processes for the uranyl dipyrrin complex **2**, highlighting ligand redox activity.

The third outer-sphere reduction, observed at *E*_1/2_ –2.02 V, could be due to either the formation of a dipyrrin-radical U(iv) complex or a U(v) complex in which the dipyrrin is doubly reduced. This third reduction process occurs at a potential similar to other U(v)/U(iv) redox couples so its assignment tends towards the former, but is more positive than those reported for related uranyl(v) Pacman complexes (Scheme 2).^56,57^ Attempts were made to isolate the product of chemical reduction of **2** with potassium metal (*E* ^o^ = *ca.* –2.4 V), to determine whether the third reduction process is ligand- or metal-based. However, this reaction did not proceed cleanly and was therefore not investigated further.

In contrast to the outer-sphere reduction chemistry of **2**, when chemical reduction was carried out with the dimeric Ti(iii) inner-sphere reductant, [TiCp_2_Cl]_2_, the paramagnetic, doubly-titanated complex **3** was observed by NMR spectroscopy and after isolation on a preparative scale. However, the Ti(iv)/Ti(iii) redox couple of [TiCp_2_Cl]_2_ is between –0.81 and –1.27 V *vs.* Fc^+^/Fc^60,61^ and is therefore similarly reducing to CoCp_2_, implying that only the dipyrrin-radical U(v) complex should be accessible. That no signal is seen in the EPR spectrum suggests that no ligand radical is present, *i.e.* that reduction to the U(iv) doubly-titanated complex **3** is favoured. This inference is also supported by the analysis of the U–O bond lengths measured by X-ray crystallography, U–O stretching frequency measured by IR spectroscopy and f–f transitions measured by NIR absorption spectroscopy, all of which are consistent with uranium adopting the +4 oxidation state in **3** (Scheme 2).

It is clear that the outer-sphere and inner-sphere reduction processes proceed by different mechanisms. The computational investigation highlights the redox non-innocence of the dipyrrin ligand and the role that coordination of the Lewis-acidic Ti centre to the uranyl oxo-group plays in promoting facile uranyl reduction, as seen recently for other uranyl-Lewis acid combinations.^20,23,30^ Therefore, the first single-electron, inner-sphere reduction occurs in two steps and results overall in an exergonic metal-based reduction by DFT calculations. In line with the evidence that the mono-reduced uranyl(v) complex **4** is, while not isolable, relatively stable against disproportionation, and the observation that no significant diamagnetic material is formed on outer-sphere reduction of **2**, the formation of **3** is found not to proceed through a higher energy disproportionation of the uranyl(v) intermediate **4**. This chemistry is similar to the reduction of uranyl ‘Pacman’ complexes in the presence of Lewis acids, although in these latter examples reduction to U(iv) has never been seen (perhaps due to the macrocyclic constraints limiting oxo-group access) and ligand-based redox activity has similarly not been observed. The outer-sphere reduction pathway is similarly exergonic according to the DFT calculations, forming sequentially mono-, di-, and trianionic compounds with ligand-based redox activity again implicit. It is apparent that the conversion of uranyl(vi) to U(iv) occurs by stepwise single-electron reduction with U(iv) favoured over U(v), and that the presence of the dipyrrin skeleton provides initial ligand-based redox activity that can assist subsequent reductions at the metal centre.

## Conclusions

The expanded Schiff-base dipyrrin acts as a tetradentate chelating ligand for the uranyl dication and, due to its low lying π*-MOs is a non-innocent redox partner in uranyl reduction. A combined study using voltammetry and EPR spectroscopy and X-ray crystallography shows that the uranyl dipyrrin complex **2** undergoes outer-sphere reduction of the dipyrrin ligand first, followed by two, sequential, one-electron reduction steps, separated by 0.85 V, likely affording a trianionic U(iv) complex at strongly cathodic potentials. Reduction of **2** by Ti(iii) results in straightforward access to the doubly titanated U(iv) product **3** *via* the uranyl(v) complex **4**, even though the Ti(iii) reagent is not sufficiently reducing based on its formal redox potential (by *ca.* 0.7 V) and further reinforces the understanding that oxo-coordination of the uranyl complex to the Lewis-acidic Ti(iii) metallocene renders the complex more susceptible to reduction. This fits well with previous results on the use of other Lewis acids to mediate [UO_2_]^2+^ reduction chemistry. Even though reduction of **2** results in the U(iv) complex **3**, DFT calculations, supported by spectroscopic analysis show that the LUMO of **2** is ligand-centred and favours a ligand-centred reduction in the first instance, followed by electron transfer to the U centre. Overall, this work shows that even though the dipyrrin ligand is involved in the electron transfer mechanism, the redox chemistry of this non-innocent system ultimately results in reduction at uranium.
