# Correction: Inner-sphere *vs.* outer-sphere reduction of uranyl supported by a redox-active, donor-expanded dipyrrin

**DOI:** 10.1039/c6sc90072k

**Published:** 2016-11-08

**Authors:** James R. Pankhurst, Nicola L. Bell, Markus Zegke, Lucy N. Platts, Carlos Alvarez Lamsfus, Laurent Maron, Louise S. Natrajan, Stephen Sproules, Polly L. Arnold, Jason B. Love

**Affiliations:** a EaStCHEM School of Chemistry , The University of Edinburgh , Joseph Black Building, David Brewster Road , Edinburgh , EH9 3FJ , UK . Email: jason.love@ed.ac.uk; b LPCNO , INSA , Université de Toulouse , 135, avenue de Rangueil , 31077 Toulouse Cedex 4 , France; c Centre for Radiochemisty Research , School of Chemistry , The University of Manchester , Oxford Road , Manchester , M13 9PL , UK; d WestCHEM School of Chemistry , University of Glasgow , Glasgow , G12 8QQ , UK

## Abstract

Correction for ‘Inner-sphere *vs.* outer-sphere reduction of uranyl supported by a redox-active, donor-expanded dipyrrin’ by James R. Pankhurst *et al.*, *Chem. Sci.*, 2016, DOI: 10.1039/c6sc02912d.



## 

In the original manuscript, the name of one of the authors was spelt incorrectly. The correct spelling of the author name ‘Carlos Alvarez Lamsfus’ has now been clarified and the complete, corrected author list is presented herein.

The Royal Society of Chemistry apologises for these errors and any consequent inconvenience to authors and readers.

