# Supplementary material for: Inner-sphere vs. outer-sphere reduction of uranyl supported by a redox-active, donor-expanded dipyrrin
Source: Chem Sci. 2016 Oct 28;8(1):108–16. doi: 10.1039/c6sc02912d (PMC5304617; doi:10.1039/c6sc02912d)
Supplement: Supplementary file 1 [file SC-008-C6SC02912D-s001.pdf]

Electronic Supplementary Material (ESI) for Chemical Science.
This journal is © The Royal Society of Chemistry 2016

Supporting Information

Inner-sphere vs. outer-sphere reduction of uranyl supported by a redox-active, donor-expanded dipyrrin

James R. Pankhurst,[a] Nicola L. Bell,[a] Markus Zegke,[a] Lucy N. Platts,[a] Carlos Alvarez Lamfsus,[b] Laurent Maron,[b] Louise S. Natrajan,[c] Stephen Sproules,[d] Polly L. Arnold,[a]* and Jason B. Love[a]*

[a] EaStCHEM School of Chemistry, University of Edinburgh, Joseph Black Building, David Brewster Road, Edinburgh, EH9 3FJ, UK

[b] LPCNO, INSA, Université de Toulouse, 135, avenue de Rangueil, 31077 Toulouse cedex 4, France

[c] Centre for Radiochemistry Research, School of Chemistry, The University of Manchester, Oxford Road, Manchester, M13 9PL, UK

[d] WestCHEM School of Chemistry, University of Glasgow, Glasgow, G12 8QQ, UK

Table of Contents

1 Experimental section 2

1.1 General methods and instrumentation 2

1.2 Synthetic procedures for isolated compounds 4

1.2.1 Synthesis of [$UO_2Cl(L)$], 2 4

1.2.2 Synthesis of [$(Cp_2TiCl)OUO(Cp_2TiCl)Cl(L)$], 3 6

1.2.3 Attempted synthesis of [$(Cp_2TiCl)OUO(L)Cl$] 4 6

1.3 Crystallographic data 8

1.4 NMR spectra 10

1.5 Infra-red spectra 15

1.6 Electronic absorption data 16

1.7 Emission data 20

1.8 Electrochemical data 24

1.9 EPR spectra 27

2 Computational section 28

2.1 Computational details 28

2.2 Cartesian coordinates for optimised geometries 29

3 References 35

1 Experimental section

1.1 General methods and instrumentation

All manipulations were carried out under a dry, oxygen-free N_2 atmosphere using standard Schlenk techniques or an MBraun Unilab glovebox. The solvents d_5-pyridine, d_8-THF and d_6-benzene were refluxed over potassium metal overnight, trap-to-trap distilled and three times freeze-pump-thaw degassed prior to use. Pyridine was degassed and refluxed over potassium metal for three days, distilled and stored over 4 Å molecular sieves. All other solvents were nitrogen-purged and dried by passage through Vacuum Atmosphere drying towers and then stored over 4 Å molecular sieves.

^{1}H NMR spectra were recorded on a Bruker AVA400 spectrometer operating at 399.90 MHz, or on Bruker AVA500 and Bruker PRO500 spectrometers operating at 500.12 MHz. ^{13}C{^{1}H} NMR spectra were recorded on Bruker AVA500 or Bruker PRO500 spectrometers operating at 125.76 MHz. ^{19}F{^{1}H} NMR spectra were recorded on a Bruker AVA400 spectrometer operating at 376.47 MHz or on a Bruker PRO500 spectrometer operating at 470.59 MHz. ^{1}H and ^{13}C{^{1}H} NMR chemical shifts are referenced to residual solvent resonances (C_6D_6: δ_H = 7.16 ppm, δ_C = 128.06 ppm; *d*$_8$-THF: δ_H = 1.73 ppm, δ_C = 25.37 ppm) calibrated against an external standard ($SiMe_4$, δ = 0 ppm). ^{19}F{^{1}H} NMR chemical shifts are referenced to an external standard (CCl_3F, δ = 0 ppm).

Single crystal X-ray diffraction data were collected using an Oxford Diffraction Supernova instrument at 120 K, fitted with a CCD area detector using CuKα radiation (λ = 1.5418 Å). Structural solution was carried out using direct methods in SHELXS-97 (Compound **2**)[1] and the *SIR*92 program (Compound **3**),[2] and were refined using a full-matrix least-square refinement on $|F|^2$ using SHELXL-97.[1] All programs were used within the *WinGX* suite.[3]

Elemental analysis was carried out by Mr. Stephen Boyer, London Metropolitan University.

Electrochemical measurements were made using an Autolab 302 potentiostat and the data processed using GPES Manager V4.9. Experiments were undertaken at room temperature in a N_2 glovebox in a 25 mL electrochemical cell with a glassy-carbon disk working electrode, a platinum gauze as the counter electrode and a silver wire as the pseudo-reference electrode. The solution employed was 1.0 mM of the compound with 0.1 M [$^{n}Bu_4N$][BPh_4] as the supporting electrolyte, in 12 cm^3 dry and deoxygenated CH_2Cl_2. All potentials were referenced against $[FeCp_2]^{+/0}$ ($E_{1/2}$, $[FeCp_2]^{+/0}$ = 0.0 V). Cyclic voltammograms were measured for quiescent solution at variable scan rates. Square-wave voltammograms were also measured for quiescent solution, using a frequency of 25 Hz, amplitude of 20 mV and step-potential of 5 mV, giving a scan-rate of 124 mV s^{-1}.

Infrared spectra were recorded on a Jasco 410 spectrophotometer as nujol mulls between NaCl disks. Intensities are assigned as: w = weak, m = medium, s = strong. UV/Vis/NIR spectra were recorded at room temperature (typically 288 K) on a Jasco V-670 spectrophotometer in a 10 mm quartz cuvette fitted with a Young's tap. Extinction coefficients were determined from at least four independent solutions of the analyte at various concentrations in the range 1 – 40 µM, using the gradient of the plot of absorbance against concentration. The intercepts of the linear fits were set at the origin.

Steady-state emission spectra were recorded in 1 cm path length quartz cuvettes appended with Young's taps (for room temperature measurements) or in 5 mm borosilicate Young's tap NMR tubes (for low temperature measurements) on an Edinburgh Instrument FP920 Phosphorescence Lifetime Spectrometer equipped with a 5 W microsecond pulsed xenon flashlamp, a 450 W continuous wave xenon lamp (with single 300 mm focal length excitation and emission monochromators in Czerny Turner configuration), a red sensitive photomultiplier in Peltier (air cooled) housing, (Hamamatsu R928P) and a removable liquid nitrogen cryostat (Edinburgh Instruments). Lifetime data were recorded following excitation with an EPL 405

picosecond pulsed diode LASER (EPL405, Edinburgh Instruments) using time correlated single photon counting (PCS900 plug-in PC card for fast photon counting). Lifetimes were obtained by both reconvolution fitting (using Ludox® as the scatterer) and tail fitting on the data obtained and found to be comparable. The qualities of fits of lifetime data were judged by minimization of residuals squared and reduced chi-squared; generally, the kinetic fits were improved using the tail fitting procedure. All spectra are reported uncorrected.

X-band EPR spectra were measured for CH_2Cl_2/toluene solutions on a Bruker ELEXSYS E500 spectrometer and simulations performed using Bruker's Xsophe software package.[4]

1.2 Synthetic procedures for isolated compounds, and attempted syntheses

The compounds HL,[5] $UO_2\{N(SiMe_3)_2\}_2(py)_2$[6] and $[Cp_2TiCl]_2$[7] were synthesised according to literature procedures. All other reagents were used as received.

1.2.1 Synthesis of $[UO_2Cl(L)]$, 2

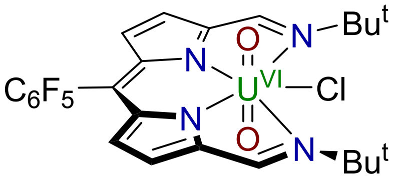

Method A: Pyridine (15 mL) was added to a stirred mixture of HL (0.40 g, 0.84 mmol) and $UO_2\{N(SiMe_3)_2\}_2(py)_2$ (0.63 g, 0.84 mmol) at −60 °C, forming a purple solution. The solution was stirred at this temperature for 2 h and at room temperature for a further 12 h, after which it had become dark blue in colour. A solution of pyridinium chloride (97.0 mg, 0.84 mmol) in pyridine (4 mL) was added to the blue solution and the mixture stirred for 12 h. The volatiles were removed under vacuum and the dried material was washed with hexanes (2 x 20 mL) and dried under vacuum for 12 h to yield $UO_2Cl(L)$, **2**. Purple-blue single crystals suitable for X-ray crystallography were obtained by re-crystallization from a concentrated benzene solution. Yield: 0.50 g (76 %). ^{1}H NMR (THF-d_8, 500 MHz): δ_H / ppm 9.52 (s, 2H, imine), 8.53 (m, 2H,

2,6-pyridine), 7.65 (m, 1H, 4-pyridine), 7.30 (d, 2H, pyrrole β-H, $^3J_{HH}$ = 4.3 Hz), 7.25 (m, 2H, 3,5-pyridine), 7.18 (d, 2H, pyrrole β-H, $^3J_{HH}$ = 4.3 Hz), 2.01 (s, 18H, ^{t}Bu); $^{13}C\{^1H\}$ NMR (THF-d_8, 126 MHz): δ_C / ppm 160.9, 159.8, 151.0, 147.6, 136.4, 135.9, 135.2, 129.2, 124.9, 124.5, 66.2, 32.7, 31.1, 23.7, 14.6; $^{19}F\{^1H\}$ NMR (THF-d_8, 471 MHz): δ_F / ppm -140.68 (m, 2F, Ar^F *o*-F), -155.19 (t, 1F, Ar^F *p*-F, $^3J_{FF}$ = 23.5 Hz), -163.34 (m, 2F, Ar^F *m*-F). Anal. Calcd for $C_{25}H_{24}ClF_5N_4O_2U$ (M_r = 780.96 g mol^{-1}): C, 38.45; H, 3.10; N, 7.17 %. Found: C, 38.40; H, 3.25; N, 7.04 %. FTIR (Nujol): υ / cm^{-1} 1655 (w, L), 1607 (w, L), 1556 (m, imine), 1408 (w, L), 1266 (m, L), 1216 (m, L), 1188 (m, L), 1062 (m, L), 1004 (m, L), 952 (w, L), 932 (m, L), 879 (w, L), 847 (s, asym. UO_2 stretch), 819 (w, L), 773 (w, L), 756 (w, L). L = absorptions attributed to the dipyrrin ligand. UV/vis (toluene): λ / nm 293sh (ε = 27,000 dm^3 mol^{-1} cm^{-1}), 513sh (6300), 557 (17,800), 598 (43,400).

Method B: A mixture of $[UO_2(N\{SiMe_3\}_2)_2(thf)_2]$ (1.536 g, 2.1 mmol, 0.5 eq.) and $[UO_2Cl_2(thf)_2]$ (1.014 g, 2.1 mmol, 0.5 eq.) was suspended in toluene (10 mL) and stirred for 10 min resulting in a dark orange suspension which was added to a red solution of HL (2 g, 4.2 mmol, 1 eq.) in toluene (10 mL). The solution immediately turned purple. After stirring for ca. 2 h the solution was filtered and the filtrate cooled overnight at -30°C yielding **2** as a crop of dark purple crystals which were isolated by filtration and dried under reduced pressure for 16 h. Yield: 1.76g (63%). ^1H NMR (C_6D_6, 500 MHz): δ_H / ppm 8.75 (s, 2H, imine), 6.67 (d, 2H, pyrrole β-H, $^3J_{HH}$ = 4.4 Hz), 6.52 (d, 2H, pyrrole β -H, $^3J_{HH}$ = 4.4 Hz), 1.92 (s, 18H, tBu); $^{13}C\{^1H\}$ NMR (C_6D_6, 126 MHz) δ_C / ppm 159.8 (C_q), 157.8 (CH), 146.7 (C_q), 146.0 (br, C-F), 144.1 (br., C-F), 138.8 (br. C-F), 136.7 (br., C-F), 134.1 (CH), 124.0 (CH), 65.7 (2 x $C(CH_3)_3$),30.7 (6 x $C(CH_3)_3$); $^{19}F\{^1H\}$ NMR (C_6D_6, 471 MHz): δ_F / ppm -138.76 (m, 2F, Ar^F *o*-F), -151.43 (t, 1F, Ar^F *p*-F, $^3J_{FF}$ = 23.5 Hz), -160.45 (m, 2F, Ar^F *m*-F);

1.2.2 Synthesis of

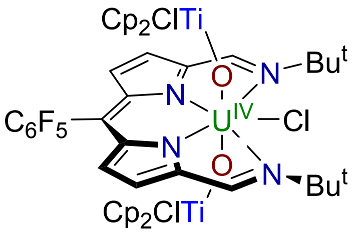

[$(Cp_2TiCl)OUO(Cp_2TiCl)Cl(L)$], 3

To a purple-blue solution of **2** (0.20 g, 0.26 mmol) in toluene (15 mL) was added a solution of $[Cp_2TiCl]_2$ (0.12 g, 0.28 mmol) in toluene (10 mL) at −40 °C. The solution was stirred for 12 h at this temperature, forming a dark blue solution which was allowed to warm to room temperature, and then stirred for a further 48 h, affording a blue precipitate. The blue solids of **3** were isolated by filtration and dried under vacuum. Single crystals suitable for X-ray diffraction were grown from a concentrated benzene solution at room temperature. Yield: 0.115 g (37 %). ^{1}H NMR (THF-d_8, 500 MHz): δ_H / ppm 43.63 (s, 20H, Cp), -17.38 (s, 2H, pyrrole β-H), -22.45 (s, 2H, pyrrole β-H), -31.68 (s, 18H, ^{t}Bu), -37.33 (s, 2H, imine); ^{19}F{^{1}H} NMR (THF-d_8, 471 MHz): δ_F / ppm -153.48 (d, 2F, Ar^F *o*-F, $^3J_{FF}$ = 20.0 Hz), -161.97 (t, 1F, Ar^F *p*-F, $^3J_{FF}$ = 22.6 Hz), -170.44 (t, 2F, Ar^F *m*-F, $^3J_{FF}$ = 20.1 Hz). Anal. Calcd for $C_{45}H_{44}Cl_3F_5N_4O_2Ti_2U$ (M_r = 1207.98 g mol^{-1}): C, 44.74; H, 3.67; N, 4.64 %. Found: C, 44.59; H, 3.69; N, 4.51 %. FTIR (nujol): υ / cm^{-1} 1659 (w, L), 1610 (m, L), 1560 (s, imine), 1521 (m, L), 1499 (s, L), 1407 (m, L), 1272 (s, L), 1220 (m, L), 1193 (s, L), 1156 (w, X), 1062 (s, L), 1001 (s, L), 982 (s, L), 962 (w, L), 948 (m, L), 898 (w), 845 (m, X), 814 (s, X), 772 (m, L), 630 (s, asym. UO_2 stretch). L = absorptions attributed to the dipyrrin ligand, X = absorptions

attributed to $TiCp_2Cl$. UV/vis/NIR (toluene): λ / nm 296sh (ε = 18,600 $dm^3\ mol^{-1}\ cm^{-1}$), 509sh (5200), 546 (10,800), 598 (11,000), 1142 (44), 1192 (12), 1380 (6), 1493 (5).

1.2.3 Attempted synthesis of [(Cp_2TiCl)OUOCl(L)], 4

To a purple-blue solution of **2** in d_8-thf (0.5 mL) was added 0.5 eq of $[TiCp_2Cl]_2$ to give a purple solution. The ^{1}H NMR spectrum taken after 5 minutes contained resonances corresponding to complex **2**, smaller resonances corresponding to complex **3** and a set of resonances which were assigned to the U^V species [Cp_2ClTiOUO(L)] (**4**, see below). After ca. 1 h microcrystalline precipitate was evident in the NMR tube. After 48 h at room temperature all three species were still present in solution. ^{1}H NMR (THF-d_8, 500 MHz): δ_H / ppm 19.35 (s, 10H, Cp), -1.12 (d, $^3J_{HH}$ = 3.6 Hz, 2H, pyrrole β-H), -3.11 (d, $^3J_{HH}$ = 3.6 Hz, 2H, pyrrole β-H), -7.84 (s, 2H, imine CH), -8.58 (s, 18H, *t*-Bu); ^{19}F{^{1}H} NMR (THF-d_8, 471 MHz): δ_F / ppm -141.35 (d, 2F, Ar^F *o*-F, $^3J_{FF}$ = 32.7 Hz), -145.82 (d, 2F, Ar^F *o*-F, $^3J_{FF}$ = 32.7 Hz), -158.00 (t, 1F, Ar^F *p*-F, $^3J_{FF}$ = 22.1 Hz), -165.96 (t, 2F, Ar^F *m*-F, $^3J_{FF}$ = 23.2 Hz), -166.24 (t, 2F, Ar^F *m*-F, $^3J_{FF}$ = 23.2 Hz).

1.2.4 Attempted synthesis of [UO_2Cl(L)][$CoCp_2$], $2^{\bullet-}$

$CoCp_2$ (23 mg, 0.12 mmol, 0.95 eq) was added to a purple-blue solution of **2** (100 mg, 0.13 mmol) in C_6D_6 (2 mL), immediately forming a magenta solution. The product was strongly paramagnetic and NMR-silent. Single crystals of $\mathbf{2}^{\bullet-}$ were grown from THF from a reaction between **2** and two equivalents of $CoCp_2$. UV/vis/NIR (toluene): λ / nm 294 (ε = 42,000 dm^3 $mol^{-1}\ cm^{-1}$), 346sh (12,000), 548 (27,000), 598 (50,000). No absorption observed in the NIR region (see spectrum below). Dilute solution of $\mathbf{2}^{\bullet-}$ in toluene has a more purple hue than that of **2**, which appears distinctly blue.

1.2.5 Attempted synthesis of $[UO_2Cl(L)][CoCp_2]_2$, $\mathbf{2}^{\bullet 2-}$

$CoCp_2$ (47 mg, 0.25 mmol, 1.95 eq) was added to a purple-blue solution of **2** (100 mg, 0.13 mmol) in C_6D_6 (2 mL), immediately forming a magenta solution. The product was strongly paramagnetic and NMR-silent. Attempts were made to grow single crystals of the product, as above, without success. UV/vis/NIR (toluene): λ / nm 311 (ε = 39,000 $dm^3\ mol^{-1}\ cm^{-1}$), 345sh (19,000), 539 (26,000), 595 (17,000). No absorption observed in the NIR region (see spectrum below). Dilute solution of $\mathbf{2}^{\bullet 2-}$ in toluene is red-purple, in contrast to that of $\mathbf{2}^{\bullet -}$, which is purple.

1.2.6 Attempted synthesis of $[UO_2(L)]K_2$

Pyridine (5 mL) was added to a mixture of **2** (36 mg, 46 μmol) and potassium metal (5 mg, 0.13 mmol, 2.8 eq), forming a dark purple solution. A multitude of paramagnetically-shifted ^1H NMR resonances were observed shortly after the reaction was started, providing no definitive structural information and indicating that a large number of products are formed from the reaction. Attempts were made, without success, to isolate single crystals from the reaction mixture.

1.3 Crystallographic data

Table S1. Summary of crystallographic data for complexes **2**, **3**, and **[CoCp$_2$][UO$_2$(L)]**.

Compound	**[UO$_2$Cl(L)] 2**	**[(Cp$_2$TiCl)OUO(Cp$_2$TiCl)Cl(L)] 3**	**[Co(Cp)$_2$][UO$_2$(L)]**
CCDC Number	1446511	1446512	1507634
Chemical formula	$C_{37}H_{36}ClF_5N_4O_2U$	$C_{51}H_{50}Cl_3F_5N_4O_2Ti_2U$	$C_{74}H_{76}Cl_2F_{10}N_8O_5Co_2U_2$
M_r / g mol^{-1}	937.18	1286.13	2012.24
Crystal system, space group	monoclinic, *Cc*	Monoclinic, $P2_1/c$	Monoclinic, $P2_1/c$
Temperature / K	120	120	170
a, b, c / Å	24.5647 (2), 8.4279 (1), 16.8859 (1)	14.486 (5), 30.377 (5), 12.092 (5)	16.0945 (2), 28.2241 (4), 19.2594 (3)
α, β, γ / deg	90.00, 91.172 (1), 90.00	90.00, 107.081 (5), 90.00	90.00, 105.247 (2), 90.00
V / Å^3	3495.14 (5)	5086 (3)	8440.7 (2)
Z	4	4	4
Radiation type	Cu *K*α	Cu *K*α	Mo *K*α
μ / mm^{-1}	14.35	13.44	4.35
Crystal size / mm	0.11 × 0.05 × 0.04	0.12 × 0.04 × 0.02	0.51 × 0.16 × 0.06
Diffractometer	SuperNova, Dual, Cu at zero, Atlas	SuperNova, Dual, Cu at zero, Atlas	Xcalibur, Eos
Absorption correction	Gaussian	Gaussian	Analytical
T_{min}, T_{max}	0.743, 0.871	0.982, 0.996	0.757, 0.950
No. of measured, independent and observed [$I > 2\sigma(I)$] reflections	34748, 7244, 7139	75069, 9330, 7021	117037, 19317, 16343
R_{int}	0.033	0.158	0.036
$(\sin \theta/\lambda)_{max}$ / Å^{-1}	0.630	0.602	0.649
$R[F^2 > 2\sigma(F^2)]$, $wR(F^2)$, S	0.027, 0.068, 1.04	0.056, 0.112, 1.06	0.033, 0.076, 1.07
No. of reflections	7244	9330	19317
No. of parameters	439	601	940
No. of restraints	2	0	60
H-atom treatment	Mixed	Mixed	Riding
$\Delta\rho_{max}$, $\Delta\rho_{min}$ / e Å^{-3}	3.81, −0.85	0.99, −1.59	1.65, -0.90

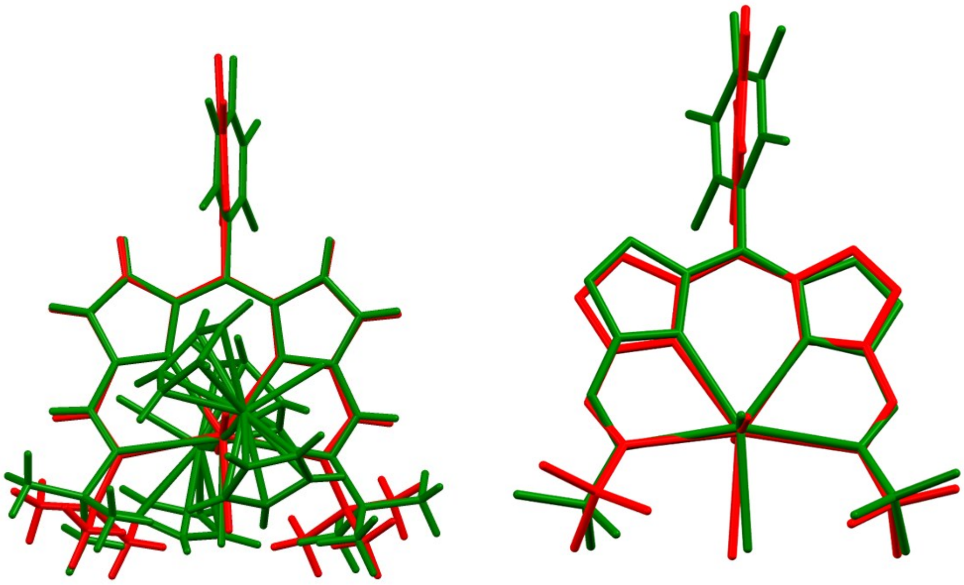

Figure S1. Overlay of X-ray crystal structures of **2** (red) and **3** (green) (left), and **2** (red) and **2**$^{\bullet-}$ (green) (right) highlighting any structural between the dipyrrin ligands in U(VI) and U(IV) complexes and in U(VI) and U(VI)(L$^{\bullet}$) complexes.

1.4 NMR spectra

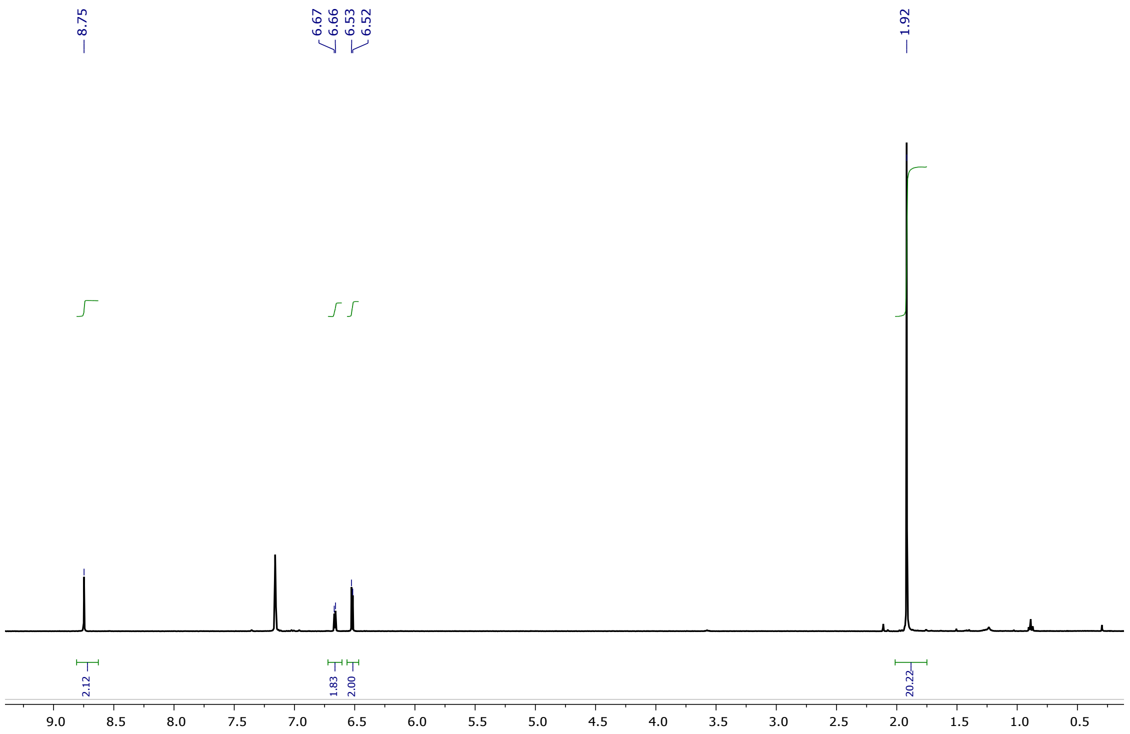

Figure S2. ^{1}H NMR spectrum for complex **2** in C_6D_6. Residual C_6D_5H solvent resonance at 7.15 ppm

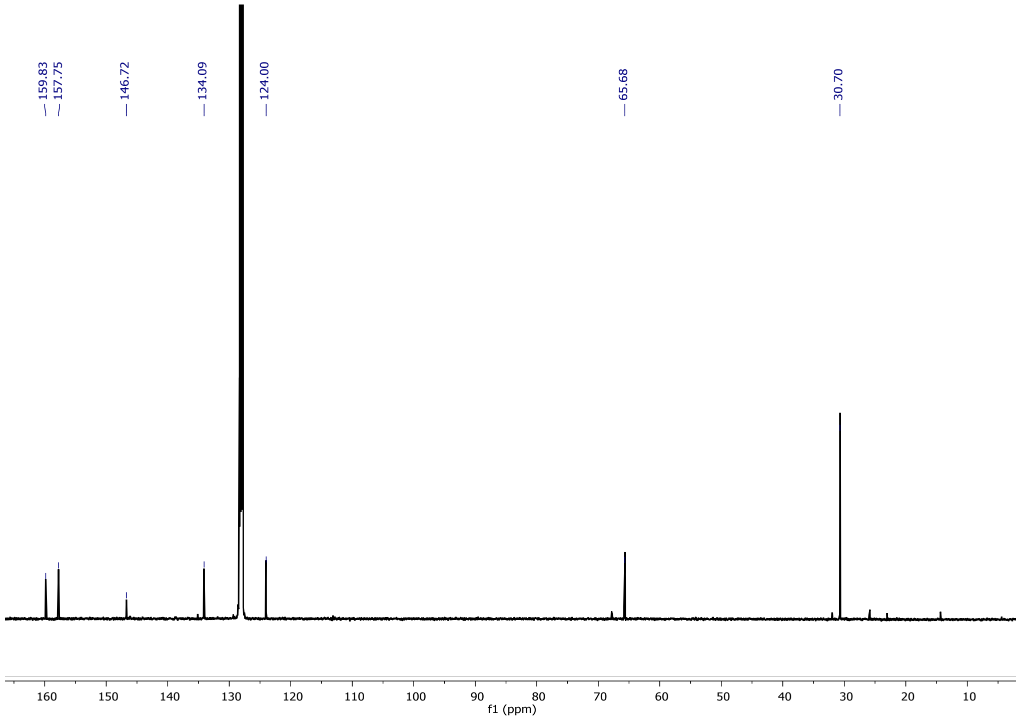

Figure S3. $^{13}C\{^{1}H\}$ NMR spectrum for complex **2** in C_6D_6

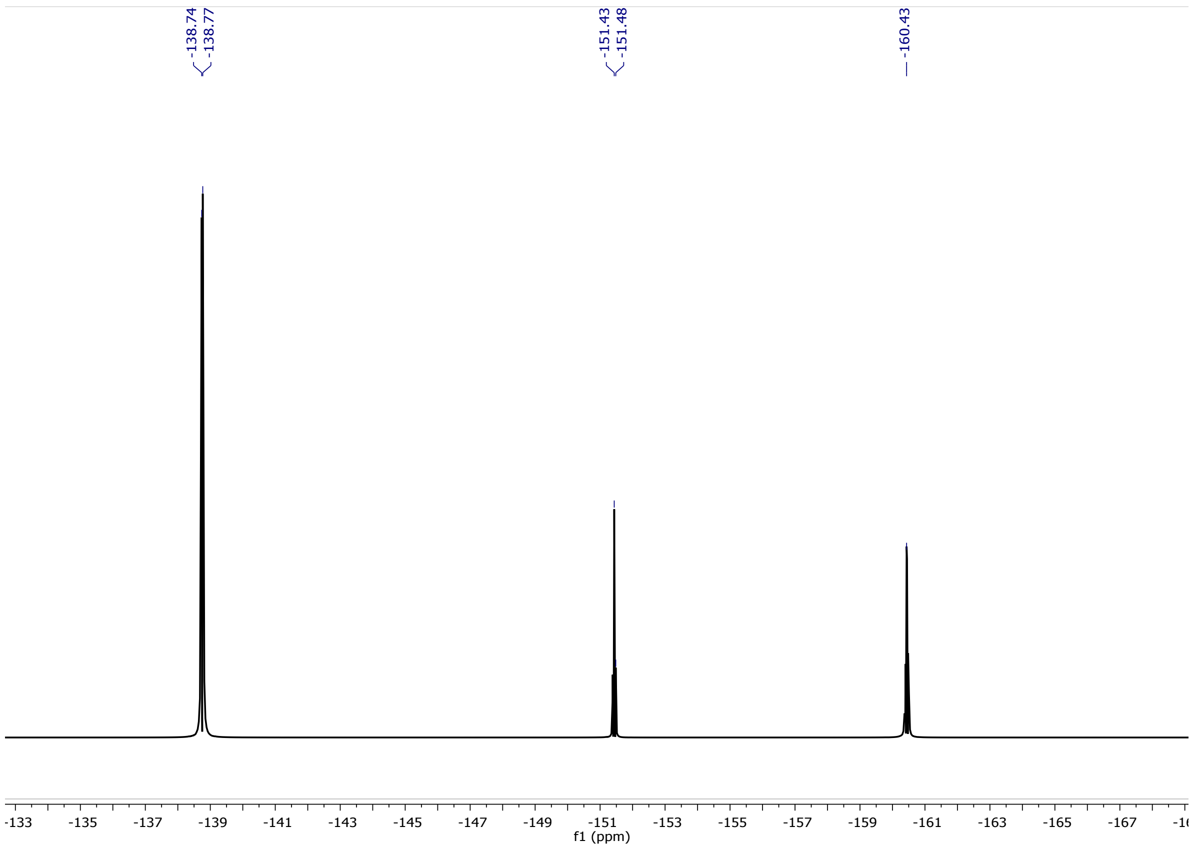

Figure S4. $^{19}F\{^{1}H\}$ NMR spectrum for complex **2** in C_6D_6.

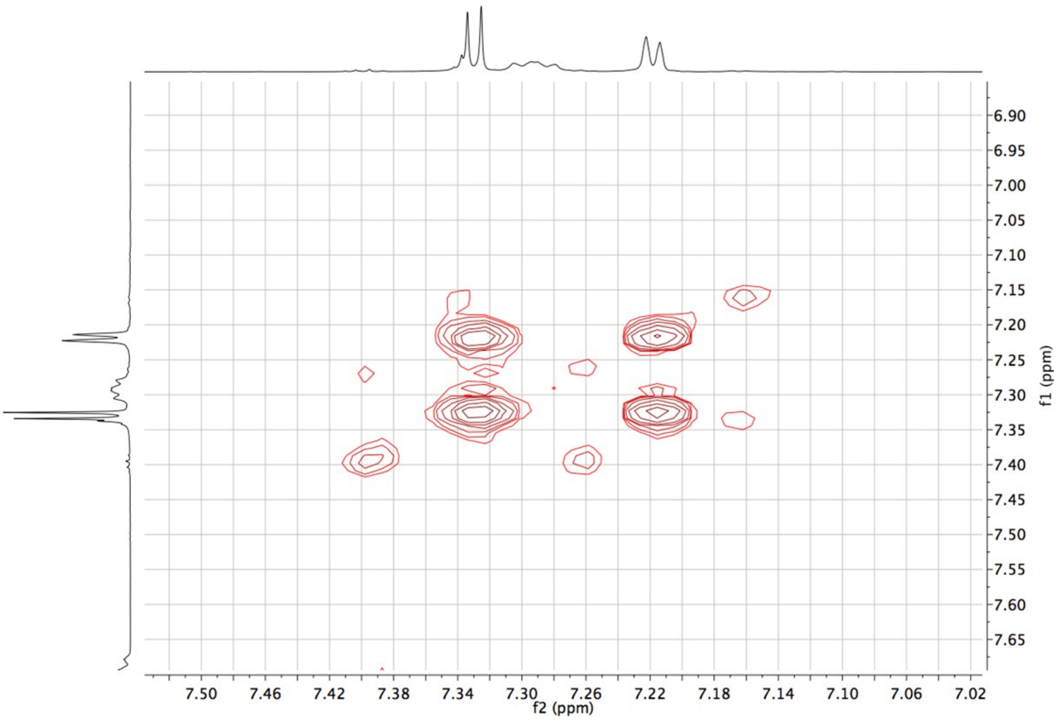

Figure S5. COSY NMR spectrum for complex **2** in d_8-THF, focussing on the pyrrole proton region.

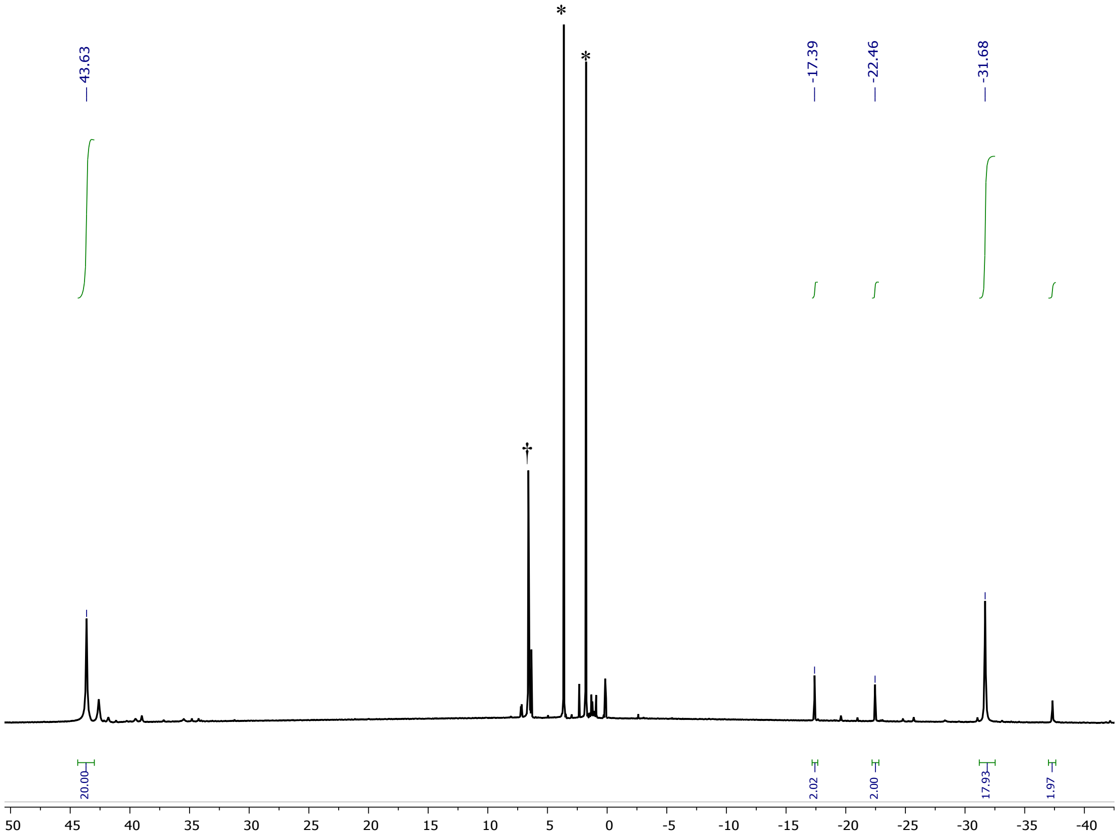

Figure S6. ^{1}H NMR spectrum for complex **3** in d_8-THF . Residual THF solvent resonance marked with an asterisk (*) and minor impurity (Cp_2TiCl_2) marked with a dagger (†).

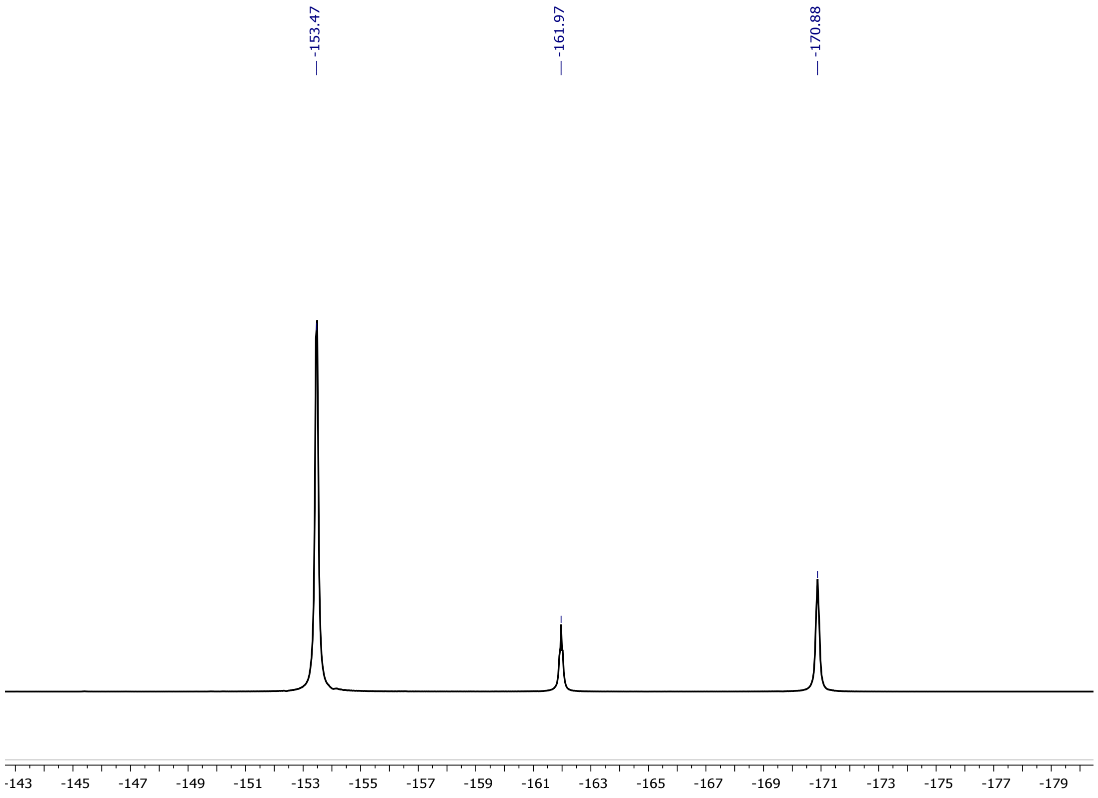

Figure S7. $^{19}F\{^{1}H\}$ NMR spectrum for complex **3** in d_8-THF.

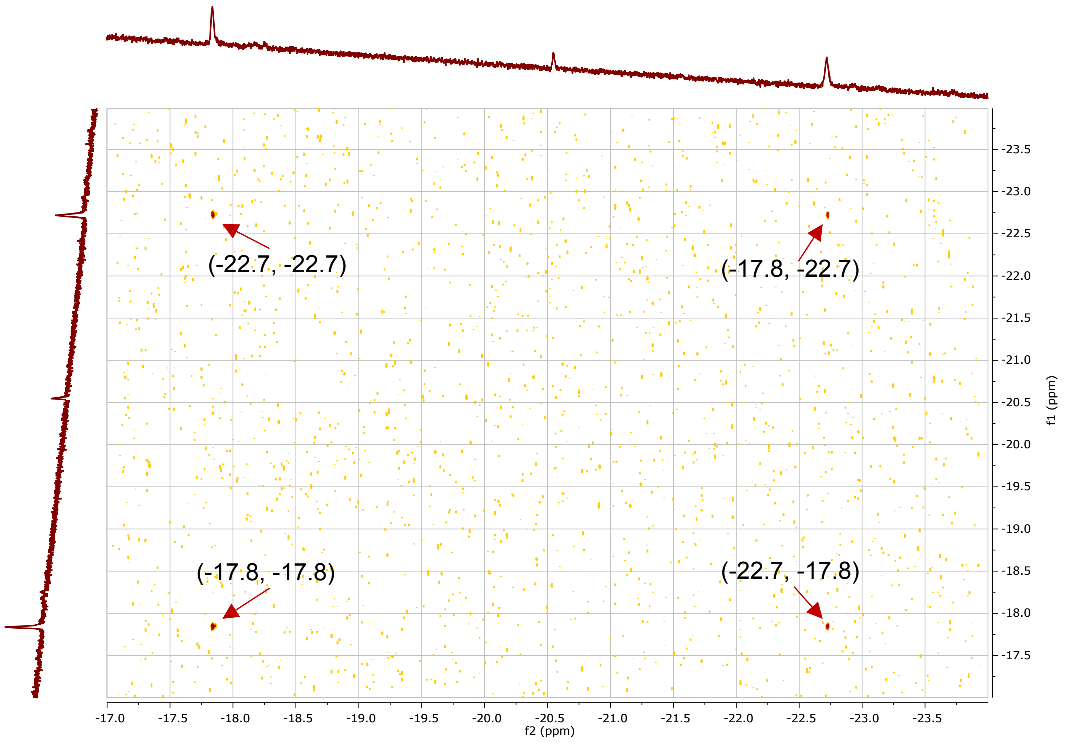

Figure S8. COSY NMR spectrum for complex **3** in d_6-benzene, focussing on the pyrrole protons.

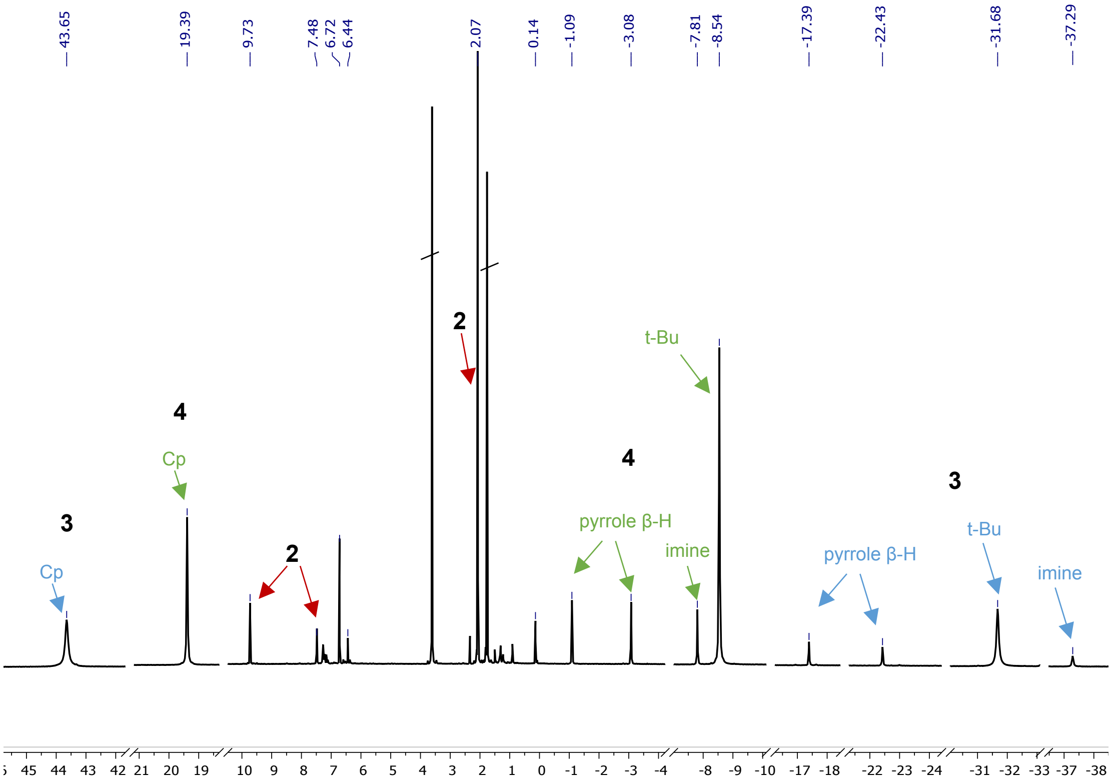

Figure S9. ^{1}H-NMR spectrum for reaction of **2** with 0.5 eq. $[TiCp_2Cl]_2$ in d_8-thf showing resonances for complex **2** (red arrows), complex **3** (blue arrows) and complex **4** (green arrows)

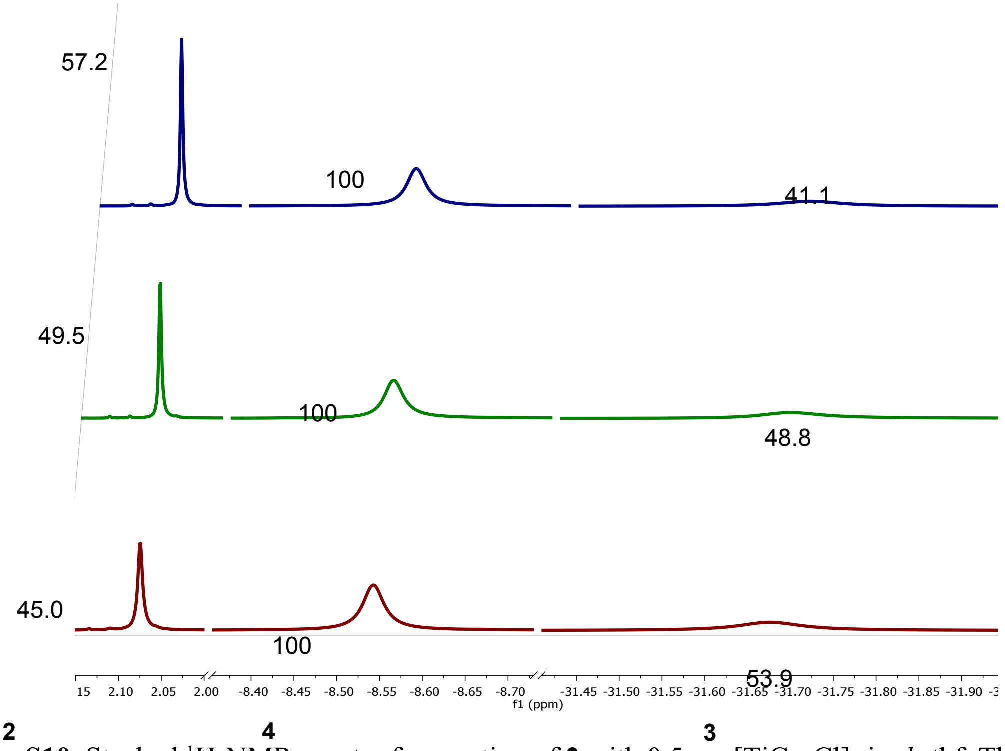

Figure S10. Stacked ^{1}H-NMR spectra for reaction of **2** with 0.5 eq. $[TiCp_2Cl]_2$ in d_8-thf. The resonances assigned toTHF showing the *t*-Bu protons for **2**, **3** and **4** are shown and their relative ratios of **2**:**4**:**3** over 24h (green) and 48h (blue) respectively. Microcrystalline precipitate in the NMR tube was assigned as complex **3**.

1.5 Infra-red spectra

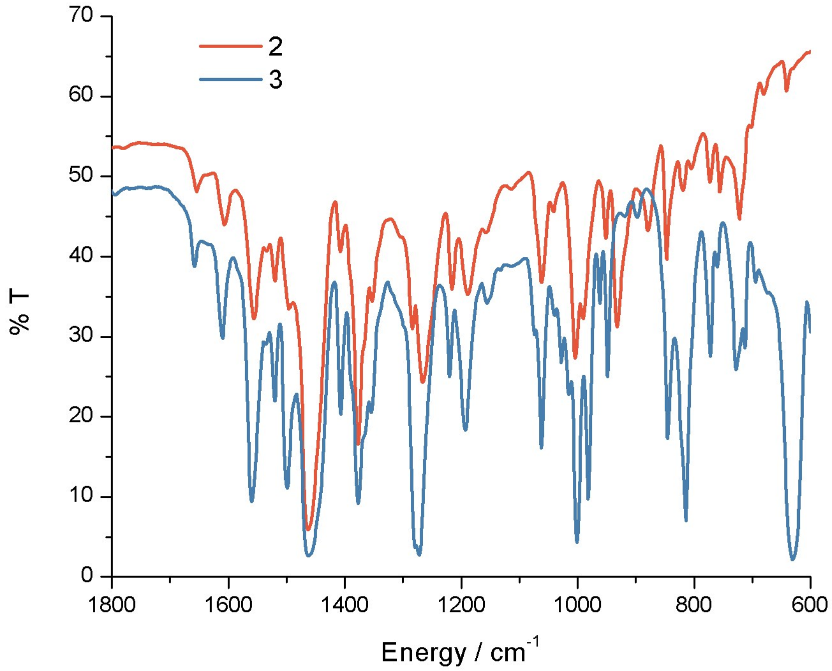

Figure S11. FT-IR spectra (nujol mull) of complexes **2** and **3**.

1.6 Electronic absorption data

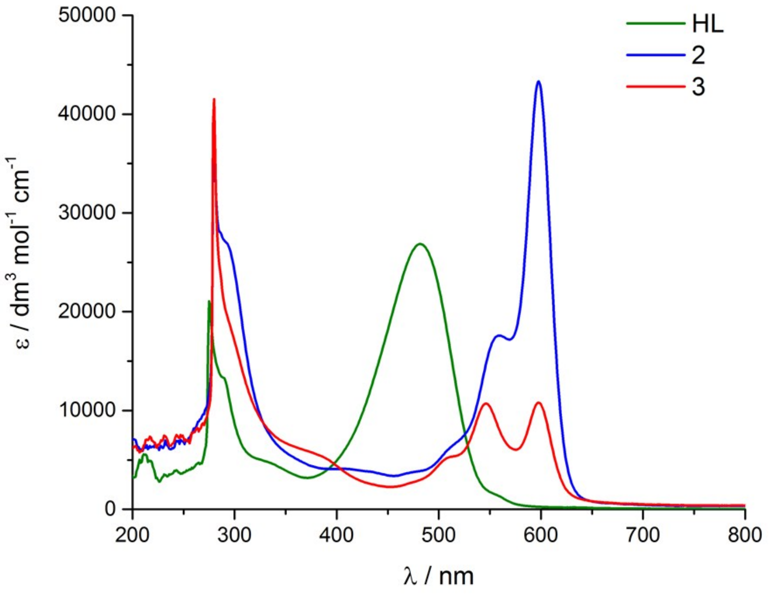

Figure S12. UV/vis spectra for HL (green), **2** (blue) and **3** (red). All measured at room temperature in toluene.

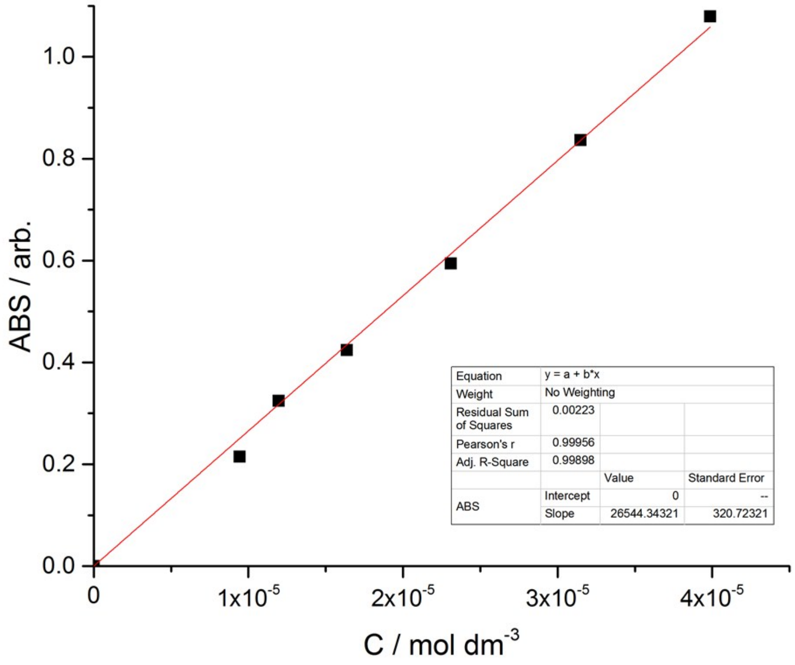

Figure S13. Determination of the extinction coefficient for HL in toluene. Absorbance values are for the maximum at 483 nm.

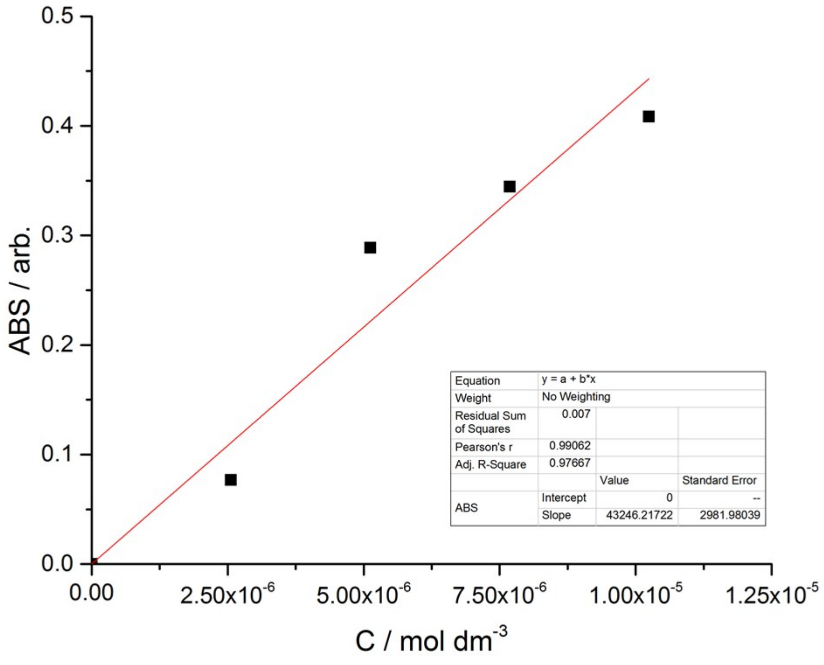

Figure S14. Determination of the extinction coefficient for **2** in toluene. Absorbance values are for the maximum at 598 nm.

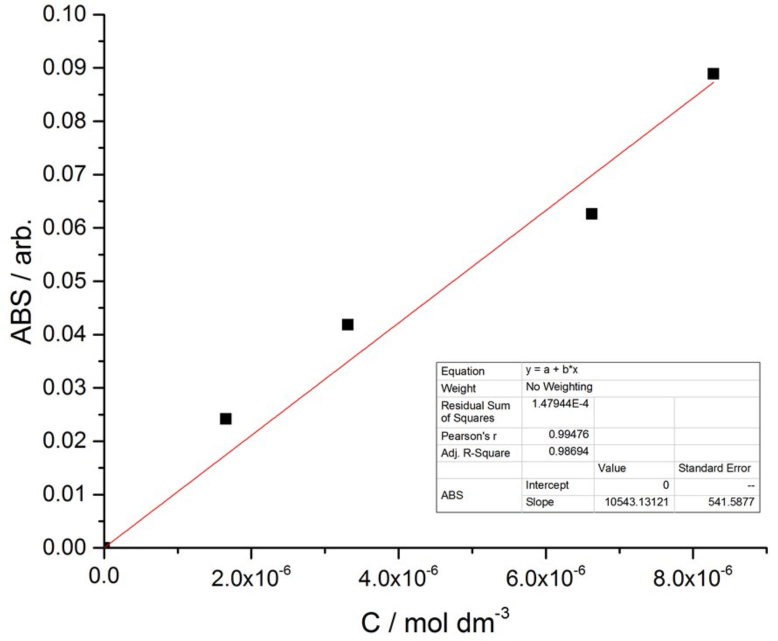

Figure S15. Determination of the extinction coefficient for **3** in toluene. Absorbance values are for the maximum at 598 nm.

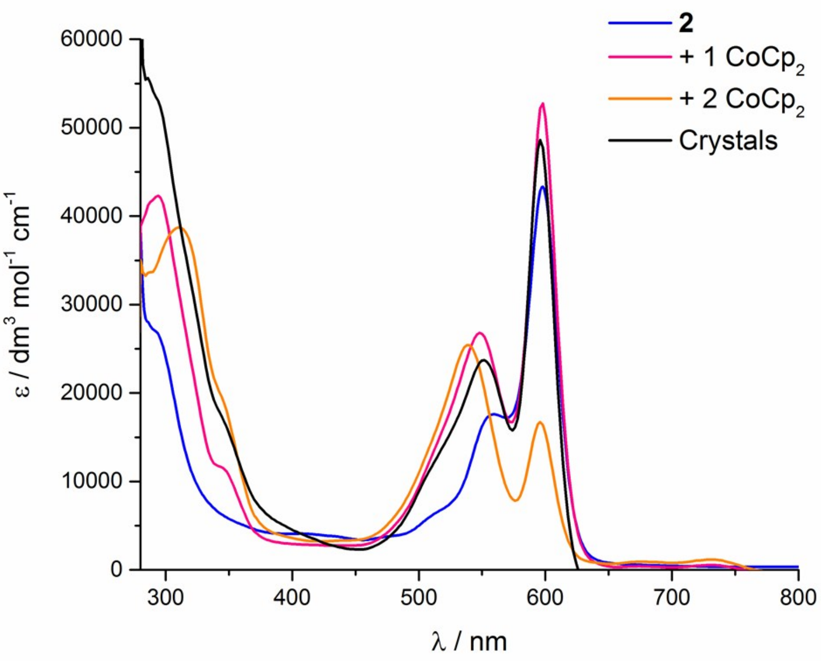

Figure S16. Overlaid UV/vis spectra for **2**, following addition of 1 and 2 eq. of $CoCp_2$, and crystalline $\mathbf{2}^{\bullet-}$. All spectra measured as toluene solutions.

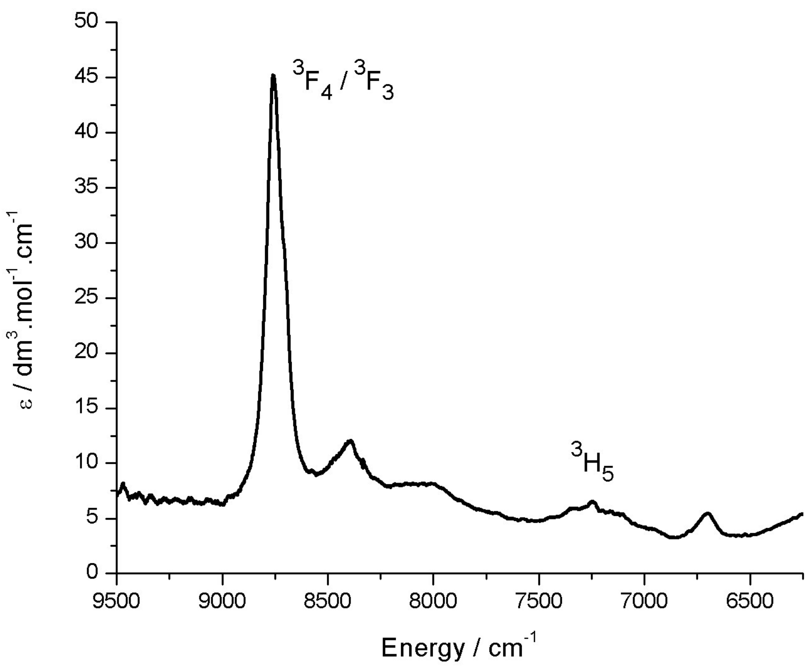

Figure S17. Room temperature NIR absorption spectrum for complex **3** (toluene solution).

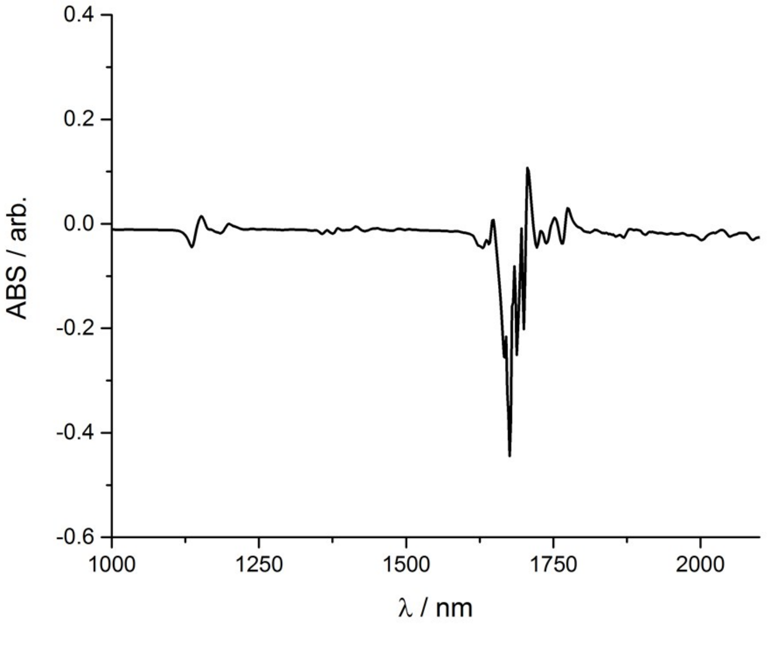

Figure S18. Room temperature NIR absorption spectrum for **2** after addition of 1 eq. $CoCp_2$ (concentrated toluene solution). Only solvent-subtraction artefacts are observed; absorption bands arising from *f*-*f* transitions are absent.

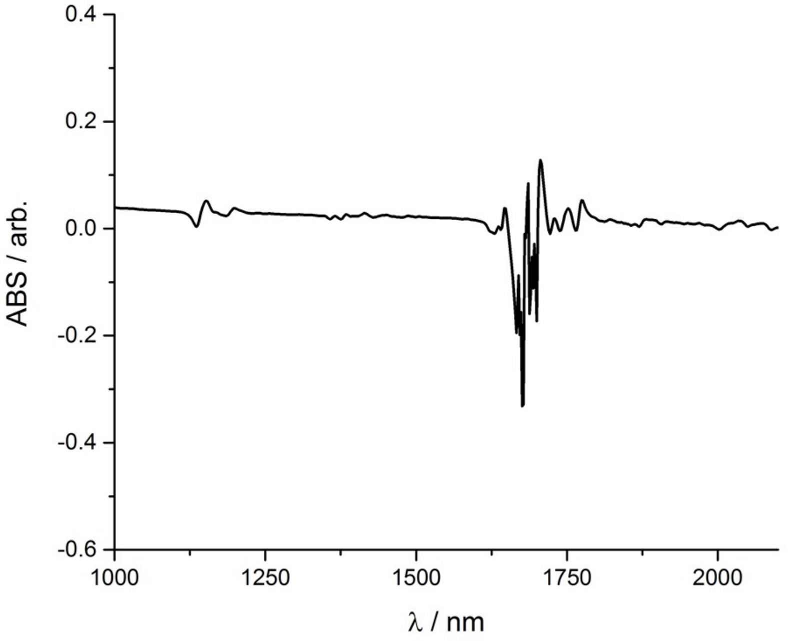

Figure S19. Room temperature NIR absorption spectrum for **2** after addition of 2 eq. $CoCp_2$ (concentrated toluene solution). Only solvent-subtraction artefacts are observed; absorption bands arising from *f-f* transitions are absent.

1.7 Emission data

Table S2. Summary of lifetime data for HL and complexes **2** and **3** in THF recorded following 405 nm excitation with a picosecond pulsed diode LASER (nd, not determined due to insufficient signal intensity at 405 nm excitation).

Compound	λ_{em} / nm	τ_1 / ns, 298K	τ_2 / ns, 298K	τ_1 / ns, 77K	τ_2 / ns, 77 K
HL	490	1.10 ± 0.02 (52%)	5.61 ± 0.08 (48%)	1.04 ± 0.06 (23%)	2.69 ± 0.04 (77%)
HL	600	0.63 ± 0.01 (54.2%)	4.69 ± 0.07 (46%)	0.51 ± 0.01 (23%)	4.12 ± 0.03 (77%)
2	490	1.33 ± 0.02 (35%)	6.98 ± 0.07 (65%)	nd	nd
2	600	1.07 ± 0.01 (57%)	4.27 ± 0.06 (43%)	nd	nd
3	550	1.08 ± 0.02 (45%)	4.67 ± 0.05 (55%)	nd	nd

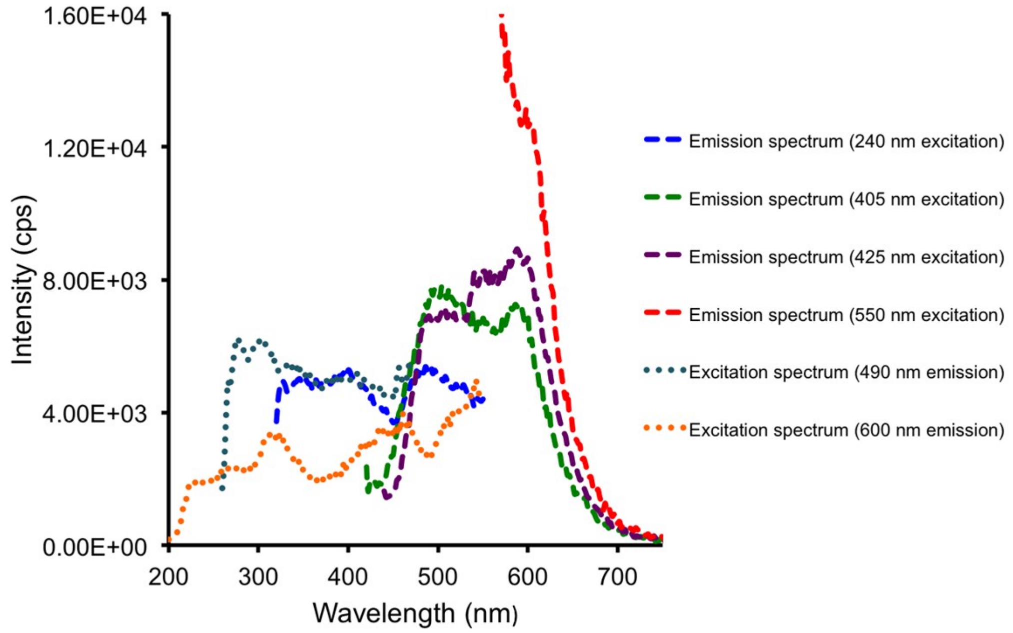

Figure S20. Emission and excitation spectra of HL in fluid THF solution recorded at 298K.

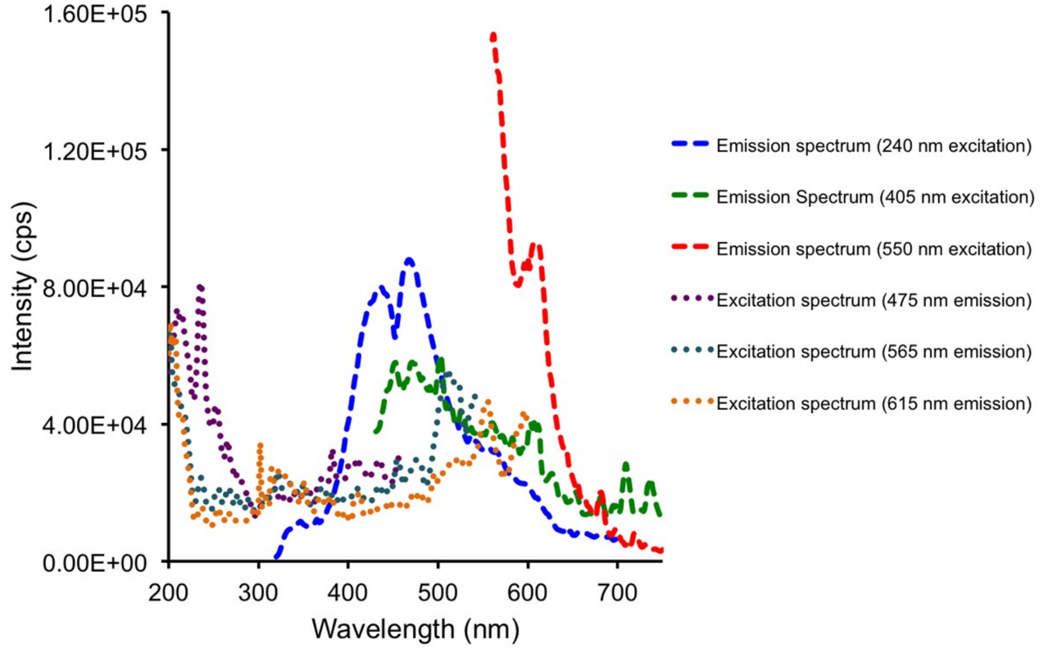

Figure S21. Emission and excitation spectra of HL in a rigid THF glass recorded at 77K.

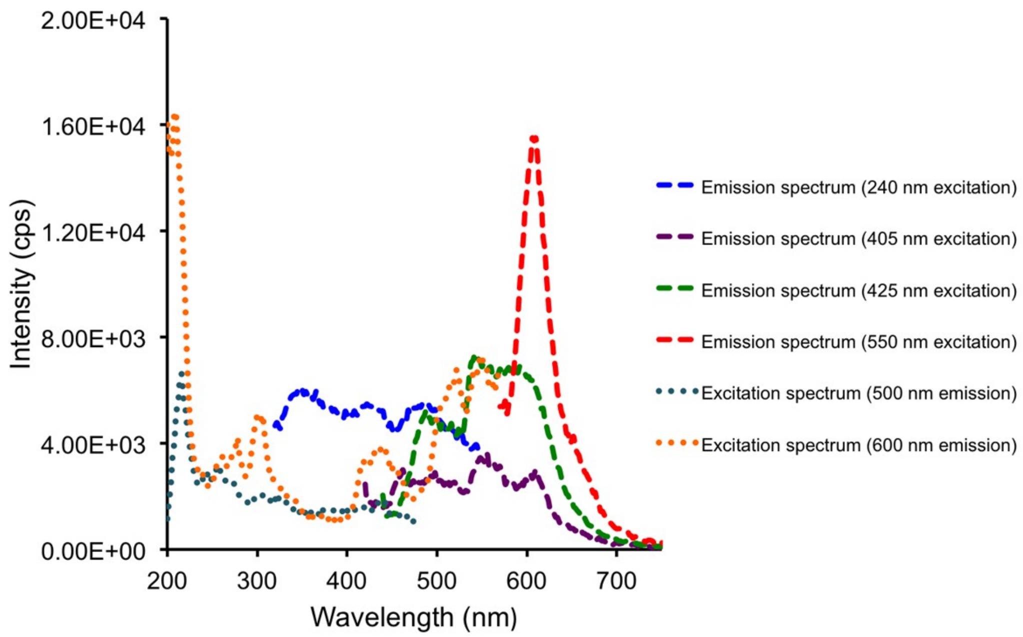

Figure S22. Emission and excitation spectra of **2** in fluid THF solution recorded at 298K.

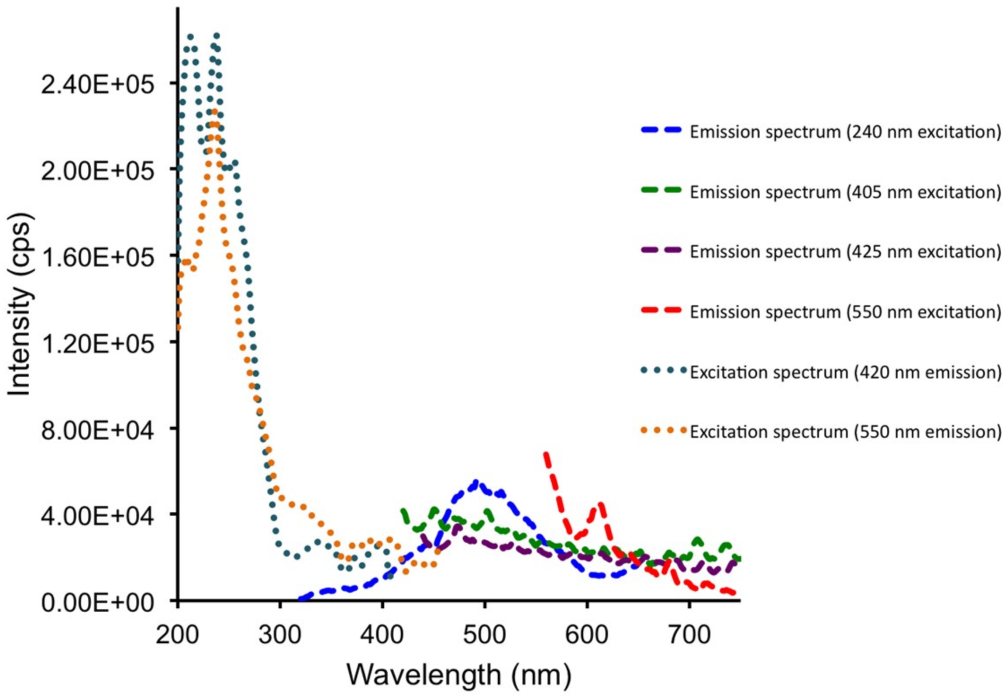

Figure S23. Emission and excitation spectra of **2** in a rigid THF glass recorded at 77K.

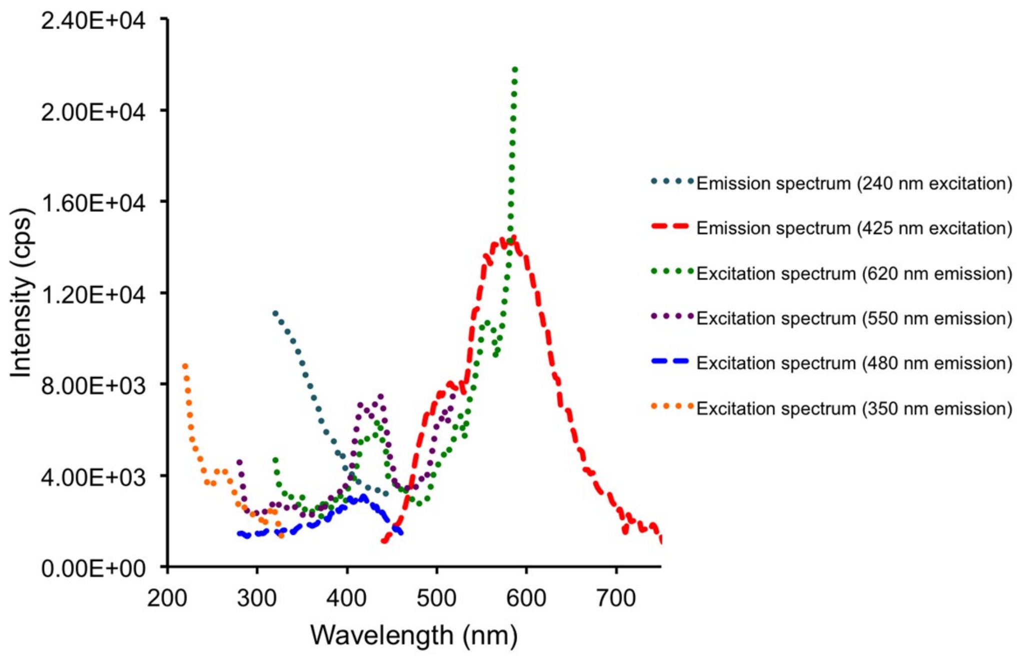

Figure S24. Emission and excitation spectra of **3** in fluid THF solution recorded at 298K.

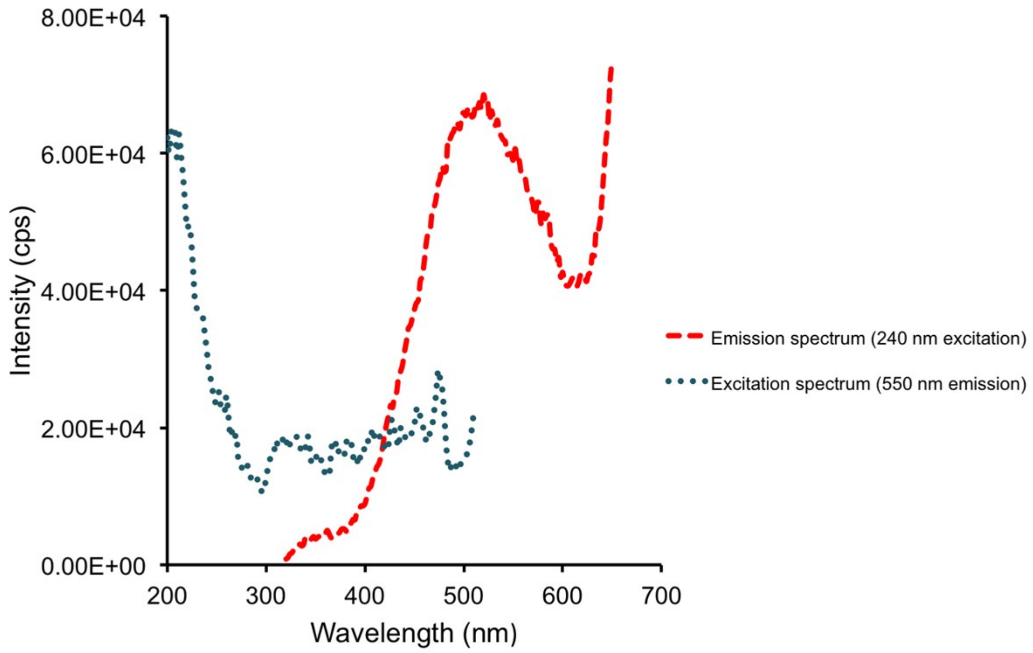

Figure S25. Emission and excitation spectra of **3** in a rigid THF glass recorded at 77K.

1.8 Electrochemical data

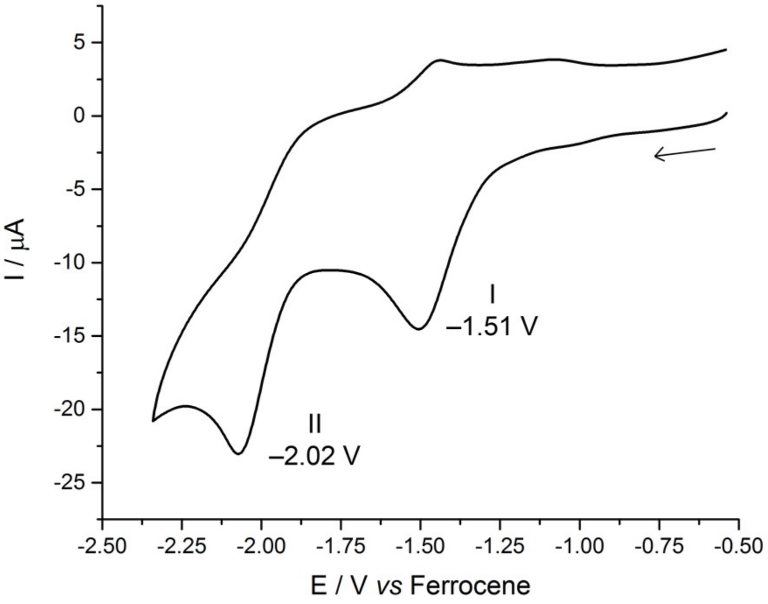

Figure S26. Cyclic voltammogram for HL, measured as a CH_2Cl_2 solution at 100 mV s^{-1}.

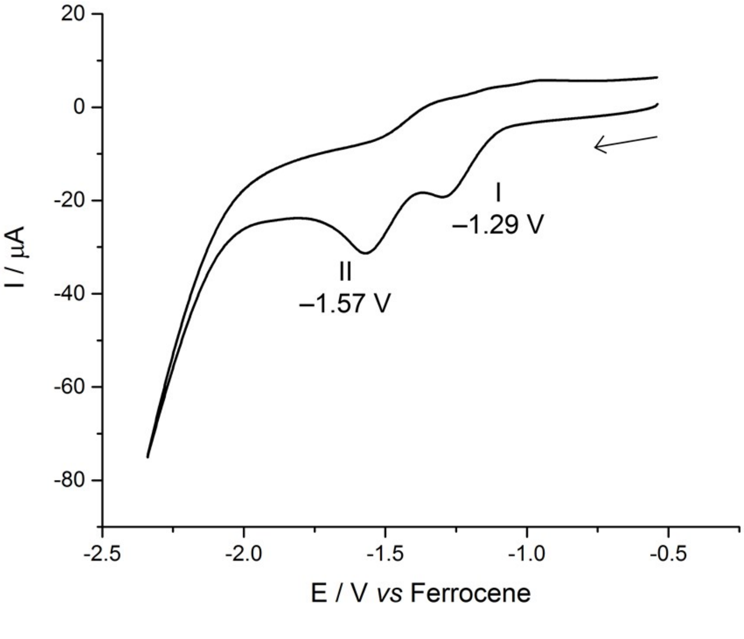

Figure S27. Cyclic voltammogram for KL, measured as a CH_2Cl_2 solution at 100 mV s^{-1}.

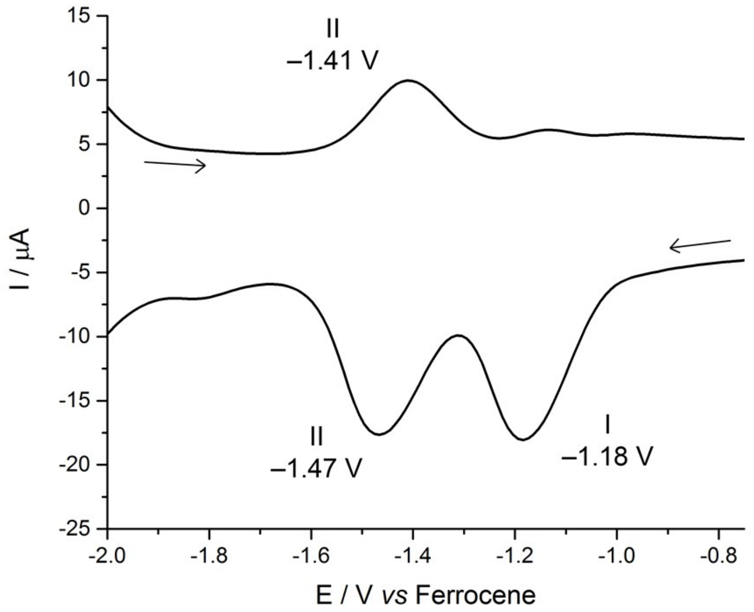

Figure S28. Square-wave voltammograms for KL, measured as a CH_2Cl_2 solution. Both the cathodic and anodic scans are shown.

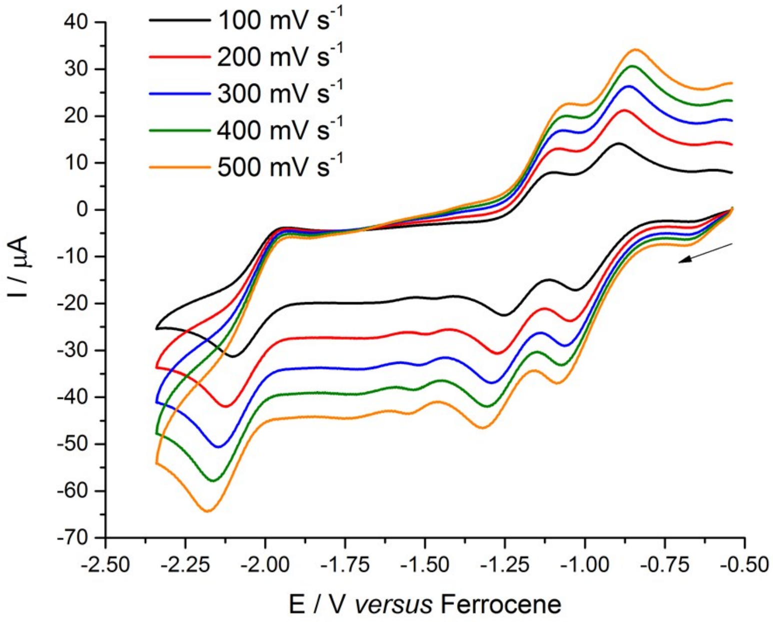

Figure S29. Cyclic voltammograms for complex **2**, measured as a CH_2Cl_2 solution at multiple scan rates between 100 – 500 mV s^{-1}.

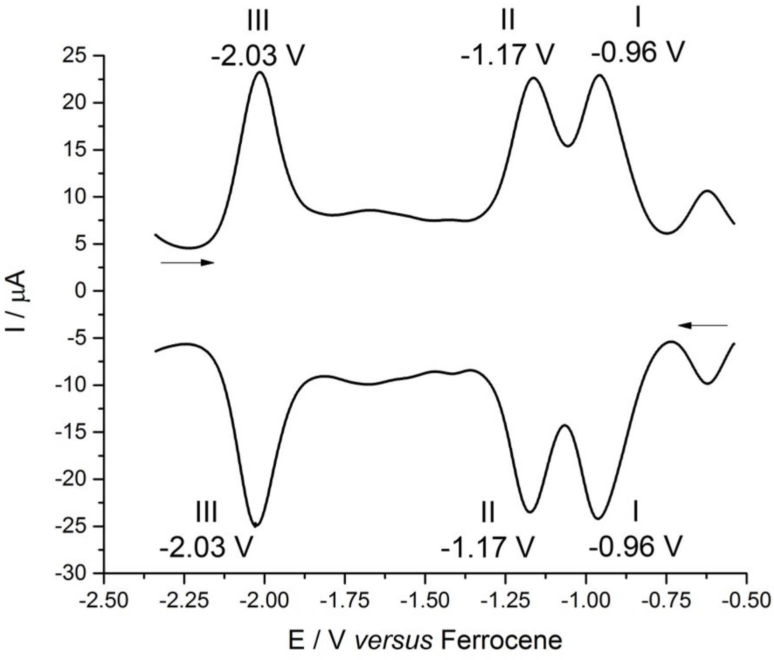

Figure S30. Square-wave voltammograms for complex **2**, measured as a CH_2Cl_2 solution. Both the cathodic and anodic scans are shown.

Table S3. Summary of cyclic voltammetry data for HL, KL and complex **2**. Values are from voltammograms measured at 100 mV s^{-1} in CH_2Cl_2 and all potentials are quoted *versus* ferrocene. [a] Irr. = irreversible, Q.R. = *quasi*-reversible, red. = reduction; * denotes poor accuracy due to overlapping waves.

Compound	Process	E_p^c / V	E_p^a / V	ΔE / V	$E_{1/2}$ / V	$\lvert i_p^r / i_p^f \rvert$	Area / µC	Assignment [a]
HL	I	–1.51	–	–	–	–	1.4E-5	Irr. 1e⁻ red.
	II	–2.02	–	–	–	–	1.3E-5	Irr. 1e⁻ red.
KL	I	–1.29	–	–	–	–	4.0E-6 *	Irr. 1e⁻ red.
	II	–1.57	–	–	–	–	6.5E-6 *	Irr. 1e⁻ red.
**2**	I	–1.03	–0.89	0.14	–0.96	1.07 *	2.8E-5	Q.R. 1e⁻ red., Dipyrrin
	II	–1.25	–1.10	0.15	–1.18	0.61 *		Q.R. U^{VI} / U^{V}
	III	–2.10	–1.94	0.16	–2.02	0.80	1.4E-5	Q.R. U^{V} / U^{IV}

Table S4. Summary of square-wave voltammetry data for KL and complex **2**. Values are from voltammograms measured at 124 mV s^{-1} in CH_2Cl_2 and all potentials are quoted *versus* ferrocene. [a] Irr. = irreversible, Q.R. = *quasi*-reversible, red. = reduction; * denotes poor accuracy due to overlapping waves.

Compound	Process	E_p^c / V	E_p^a / V	ΔE / V	$E_{1/2}$ / V	$\lvert i_p^r / i_p^f \rvert$	Area / µC	Assignment [a]
KL	I	–1.18	–	–	–	–	1.6E-6	Irr. $1e^-$ red.
	II	–1.47	–1.41	0.06	–	0.59	1.5E-6	Irr. $1e^-$ red.
**2**	I	–0.96	–0.96	0	–0.96	0.93 *	5.4E-6	Q.R. $1e^-$ red., L
	II	–1.17	–1.17	0	–1.17	0.95 *		Q.R. U^{VI} / U^{V}
	III	–2.03	–2.03	0	–2.03	0.96	2.6E-6	Q.R. U^{V} / U^{IV}

1.9 EPR spectra

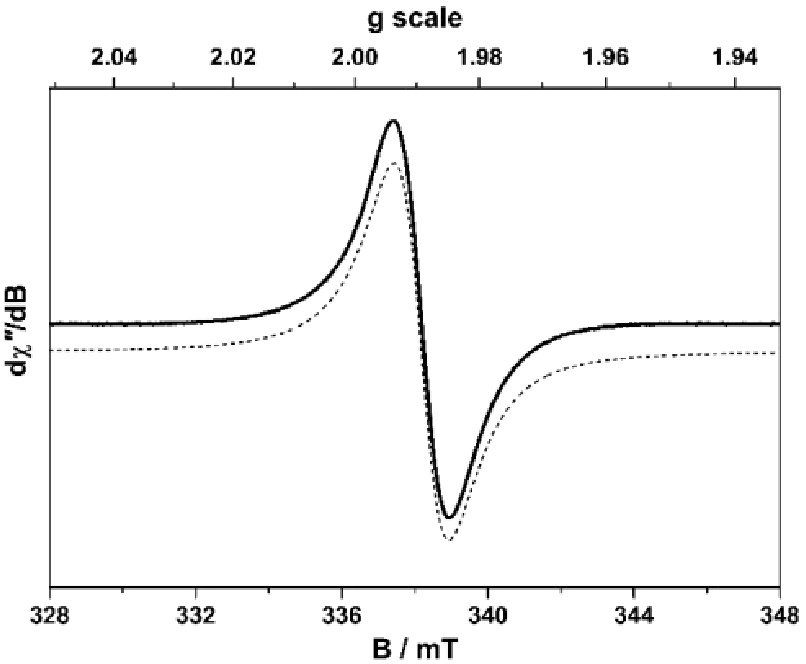

Figure S31. X-band EPR spectrum of [**2**]$^-$ recorded in fluid toluene/CH_2Cl_2 solution at 223 K, following *in situ* chemical reduction of **2** with 0.9 $CoCp_2$. Experimental conditions: frequency, 9.4155 GHz; power, 6.3 mW; modulation, 0.05 mT. Experimental data are represented by the black line; simulation is depicted by the dashed trace: g_{iso} = 1.9893.

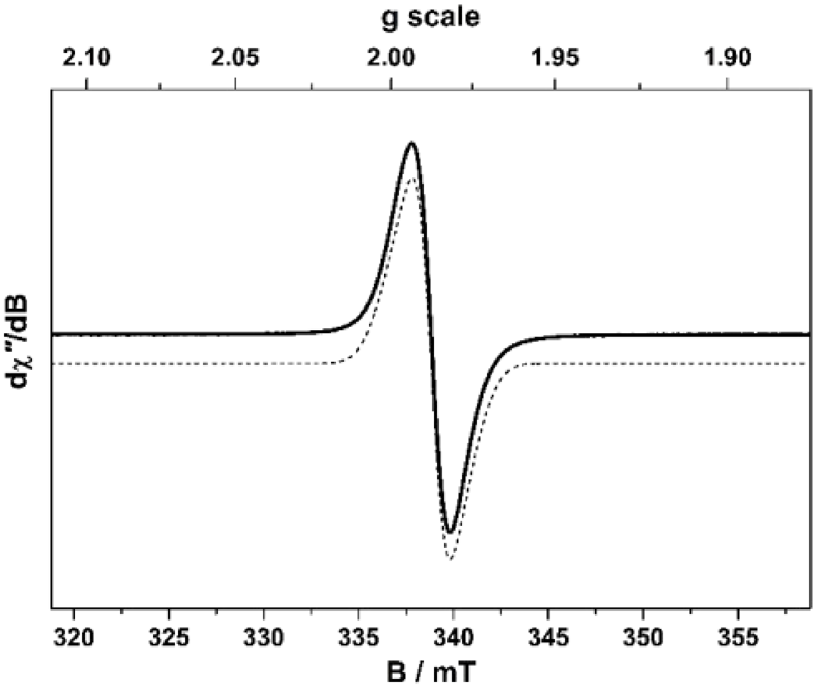

Figure S32. X-band EPR spectrum of [**2**]$^{-}$ recorded in frozen toluene/CH_2Cl_2 solution at 130 K, following *in situ* chemical reduction of **2** with 0.9 $CoCp_2$. Experimental conditions: frequency, 9.4252 GHz; power, 0.63 mW; modulation, 0.1 mT. Experimental data are represented by the black line; simulation is depicted by the dashed trace: g = (1.9974, 1.9872, 1.9786).

2 Computational section

2.1 Computational details

All quantum-chemical calculations were conducted using the Gaussian09 program suite.[8] Becke's 3-parameter hybrid functional was employed, combined with the non-local correlation functional provided by Perdew/Wang, denoted as B3PW91.[9] Two different effective core potentials from Stuttgart-Dresden were used for describing the uranium atoms. The relativistic energy-consistent small-core pseudopotential of the Stuttgart-Köln ECP library was used in combination with its adapted segmented basis.[10] For comparison, the corresponding 5*f*-in-core ECP augmented by a *f* polarization function was used.[11] It should be noted that this computational scheme was also applied previously by our group in analogous studies.[12] Ti and Cl centers were also treated with an energy-consistent pseudopotential of the Stuttgart-Köln ECP library in combination with its adapted segmented basis.[13] For all other atoms, a standard

6-31G** basis set was used.[14] All stationary points have been identified as minima (number of imaginary frequencies $N_{imag} = 0$) by frequency calculations.

2.2 Cartesian coordinates for optimised geometries

Uranium (IV)

106			
C	-1.387018	6.676304	8.709378
H	-1.735668	7.704356	8.853532
H	-0.663132	6.447749	9.491123
H	-2.241649	5.999612	8.801935
C	0.441059	7.472915	7.133483
H	0.833715	7.389478	6.114434
H	1.233585	7.240305	7.844562
H	0.131347	8.510334	7.295073
C	-1.825049	6.934523	6.275502
H	-1.468986	6.811532	5.246245
H	-2.056386	7.993965	6.415412
H	-2.764918	6.385844	6.397851
C	-0.762623	6.540423	7.313987
C	-0.889007	4.370221	6.357645
H	-1.768839	4.709040	5.812539
C	-0.483301	3.020176	6.059054
C	-1.122347	2.165666	5.121370
H	-2.015997	2.395122	4.557062
C	-0.377727	1.012922	5.101178
H	-0.555438	0.123977	4.512161
C	0.693486	1.197431	6.033253
C	1.686756	0.250856	6.312821
C	1.672183	-0.999751	5.497617
C	2.092131	-1.002412	4.165815
C	2.085342	-2.161775	3.396406
C	1.646876	-3.356557	3.959010
C	1.221320	-3.384222	5.283540
C	1.239557	-2.213598	6.035590
C	2.694589	0.362986	7.277482
C	3.756454	-0.578554	7.471127
H	3.910023	-1.482417	6.898240
C	4.528800	-0.097431	8.498437
H	5.425293	-0.530088	8.921287
C	3.914733	1.117914	8.905282
C	4.351859	1.938076	10.007096
H	5.238495	1.571182	10.521293
C	4.274648	3.698073	11.599472
C	5.345319	2.899337	12.360284
H	4.986091	1.912453	12.673844
H	5.599981	3.455880	13.266306
H	6.272723	2.779584	11.790277

C	3.093393	3.927616	12.549518	H	-3.967722	2.618015	12.071625
H	2.293614	4.484482	12.061304	C	-1.935967	2.296690	12.927266
H	3.426831	4.501800	13.419865	H	-2.057285	1.406728	13.528545
H	2.700829	2.968400	12.903878	C	-0.717575	2.995316	12.720246
C	4.903668	5.020287	11.139534	H	0.246081	2.730379	13.133872
H	5.718092	4.819347	10.437523	C	-0.996003	4.125441	11.911887
H	5.308023	5.551809	12.007497	H	-0.268386	4.837649	11.548523
H	4.166715	5.662062	10.657955	C	-2.352234	4.077412	11.558023
N	-0.257388	5.138312	7.170388	H	-2.862457	4.760277	10.895983
N	0.601562	2.450551	6.612258	C	-0.066096	0.063846	10.234052
N	2.819242	1.402902	8.181093	H	0.997425	0.172363	10.079466
N	3.737101	2.998837	10.389121	C	-1.054056	0.115845	9.233803
O	0.198062	2.760917	9.847711	H	-0.888402	0.270943	8.177786
O	3.305908	4.443883	7.167339	C	-2.323480	-0.000826	9.862846
F	2.526195	0.117339	3.605244	H	-3.280237	0.038073	9.362471
F	2.495137	-2.135594	2.137398	C	-2.118898	-0.103169	11.246103
F	1.634662	-4.463471	3.236631	H	-2.894174	-0.187676	11.994872
F	0.799863	-4.520267	5.817891	C	-0.715375	-0.024927	11.486828
F	0.817703	-2.270052	7.290757	H	-0.228324	-0.068218	12.452164
Cl	-2.811777	2.810952	8.851086	C	4.551951	6.874437	7.528853
Cl	6.312956	3.536904	7.666899	H	3.846138	6.857786	8.347213
Cl	1.755676	5.969028	10.015635	C	5.909851	6.528728	7.599996
Ti	-1.300861	2.102934	10.587013	H	6.443491	6.224244	8.487447
Ti	4.814786	4.806146	6.263642	C	6.460235	6.593855	6.288164
U	1.732827	3.676552	8.535469	H	7.480790	6.356291	6.018012
C	-2.939119	2.936821	12.178235	C	5.433070	6.998245	5.415750

H	5.525105	7.158154	4.350743
C	4.237171	7.121215	6.169719
H	3.262984	7.382895	5.779330
C	5.633756	4.443617	3.988149
H	6.401100	5.088860	3.583410
C	4.227475	4.681117	3.960564
H	3.729922	5.525488	3.501669
C	3.591480	3.583945	4.585920
H	2.529305	3.480924	4.752803
C	4.589853	2.717717	5.066271
H	4.435938	1.831410	5.664233
C	5.853448	3.246917	4.683204
H	6.815607	2.824160	4.934050

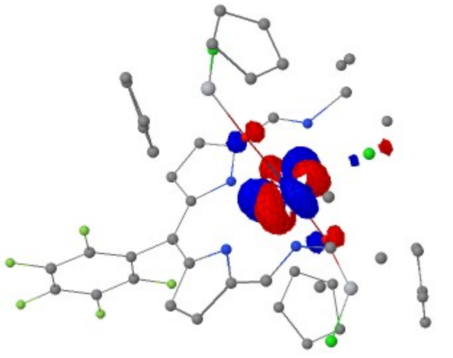

HOMO

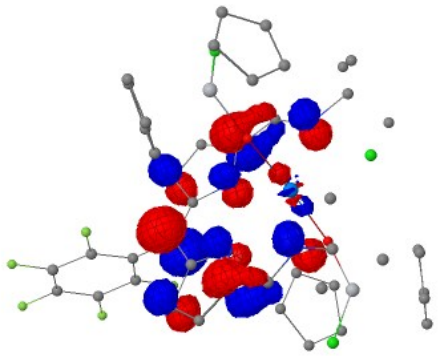

LUMO

Uranium (V)

84

O	2.771222	7.239600	9.277755
U	3.927329	6.222865	10.312360
Cl	6.079005	7.800000	10.652282
N	2.143156	5.190874	11.659660
C	1.760328	5.821086	12.782934
C	0.898110	5.020231	13.580255
C	0.762113	3.836884	12.902535
C	1.529960	3.954956	11.698409
C	2.152540	7.182256	13.034180
N	3.008732	7.794711	12.295620
C	3.219525	9.259368	12.540306
C	2.071543	9.907112	13.331888
C	1.599544	3.000755	10.680388
C	2.334758	3.119223	9.489243
C	2.289020	2.200464	8.394470
C	3.124690	2.695930	7.421759
C	3.659581	3.897746	7.951932
N	3.186128	4.160188	9.185408
C	4.591200	4.779320	7.302866
N	5.096456	5.805790	7.889641
C	5.995350	6.696671	7.090514

C	5.445466	8.125485	7.207214	C	7.726985	5.163861	13.265697
C	4.523338	9.435365	13.332864	C	6.493855	5.743370	13.649623
C	3.300404	9.947530	11.171973	C	5.876793	4.885785	14.570652
C	0.800741	1.754775	10.854982	H	4.703746	10.502259	13.500193
C	-0.596695	1.776874	10.838400	H	5.376417	9.026073	12.791052
C	-1.353716	0.621813	11.004746	H	4.450642	8.945937	14.310255
C	-0.712416	-0.598309	11.192681	H	2.392319	9.767970	10.591018
C	0.677443	-0.654026	11.205325	H	4.151882	9.587674	10.594408
C	1.417014	0.512257	11.033046	H	3.420868	11.025747	11.318994
F	-1.243422	2.917957	10.638975	H	1.099473	9.730358	12.859105
F	-2.677277	0.676077	10.977553	H	2.238523	10.987316	13.351572
F	-1.421586	-1.702643	11.350242	H	2.026144	9.580237	14.376364
F	1.289193	-1.815986	11.376306	H	1.641539	7.679286	13.858573
F	2.735863	0.413961	11.041676	H	0.459196	5.301777	14.527760
C	6.062280	6.334952	5.599132	H	0.198113	2.967067	13.207117
C	7.409334	6.583599	7.679407	H	1.690374	1.301604	8.349295
O	5.085791	4.815407	11.499420	H	3.341903	2.273700	6.449936
Ti	6.057645	3.657684	12.487466	H	4.831986	4.529972	6.270094
C	6.212530	1.376204	11.613503	H	6.509971	5.351946	5.415184
C	5.884420	2.228274	10.534508	H	6.701007	7.070082	5.102407
C	6.967802	3.112313	10.332253	H	5.080029	6.377418	5.115738
C	7.957626	2.819054	11.297121	H	7.418020	6.825054	8.742649
C	7.487961	1.737762	12.091153	H	8.076186	7.282341	7.163992
Cl	4.207146	2.538974	13.517869	H	7.803696	5.571468	7.536037
C	6.758016	3.789912	14.815403	H	4.422817	8.177808	6.824064
C	7.910325	3.983086	14.040616	H	6.076400	8.803375	6.623094

H	5.443827	8.470190	8.240790
H	6.547915	2.943958	15.455478
H	8.766609	3.324375	13.999396
H	8.423124	5.584490	12.552028
H	6.072382	6.635482	13.213245
H	4.887031	4.996279	14.989536
H	7.010217	3.918534	9.613646
H	4.941287	2.241166	10.009023
H	5.570264	0.617926	12.035819
H	8.003916	1.279869	12.924068
H	8.908781	3.323231	11.400488

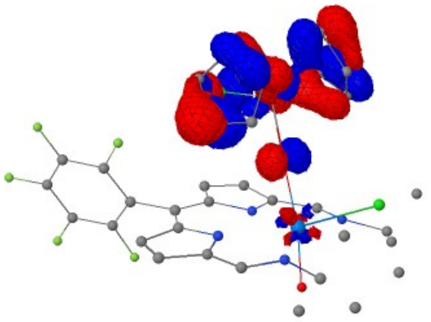

HOMO

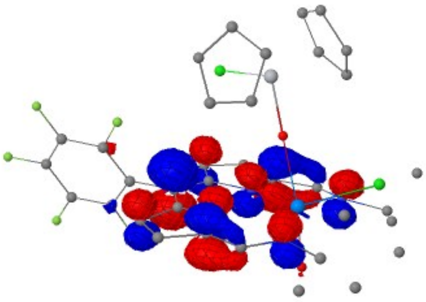

LUMO

Uranium (VI)

62

C	13.563311	2.826943	4.883827
H	12.671488	2.334624	5.286323
H	14.321475	2.060488	4.693319
H	13.309028	3.302796	3.936911
C	15.397219	4.517445	5.396811
H	15.235536	5.036567	4.452590
H	16.166843	3.754911	5.240428
H	15.766370	5.234907	6.136267
C	14.434854	3.105161	7.201050
H	14.797730	3.779439	7.984912
H	15.234265	2.389488	6.991665
H	13.587976	2.528281	7.588942
C	14.109739	3.846019	5.894996
C	12.469216	5.010554	7.171152
H	12.661791	4.317794	7.990106
C	11.493624	6.033273	7.441422
C	10.678723	6.127926	8.602893
H	10.686723	5.448430	9.444429
C	9.896208	7.239152	8.416537
H	9.148893	7.640204	9.086739

C	10.267493	7.797024	7.150652	C	13.429293	7.861834	0.191828
C	9.734862	8.966892	6.599398	H	13.245101	6.863698	0.588765
C	8.645880	9.638122	7.371406	H	14.186741	7.788051	-0.595262
C	8.919091	10.688469	8.248044	H	12.505520	8.242981	-0.256798
C	7.912710	11.318278	8.973832	C	15.249759	8.351007	1.901690
C	6.594848	10.896178	8.827363	H	15.576231	9.043244	2.683844
C	6.293028	9.850822	7.960015	H	16.018524	8.322224	1.122856
C	7.316121	9.234786	7.245217	H	15.161366	7.352936	2.329517
C	10.134784	9.580756	5.408792	N	13.081319	4.920316	6.046248
C	9.605194	10.808614	4.894321	N	11.258723	7.030265	6.573287
H	8.813139	11.390945	5.343965	N	11.133074	9.116319	4.576896
C	10.302269	11.088989	3.746865	N	12.902601	8.820452	2.381467
H	10.187216	11.936419	3.084633	O	10.941044	6.350839	3.765785
C	11.224851	10.018445	3.587122	O	13.834461	7.643908	4.999712
C	12.183879	9.874677	2.524074	F	10.166979	11.107614	8.411002
H	12.275093	10.731175	1.856286	F	8.200039	12.311865	9.801333
C	13.923302	8.818817	1.287173	F	5.630778	11.487681	9.511953
C	14.156055	10.200649	0.656537	F	5.038465	9.450650	7.820060
H	13.287515	10.573529	0.102612	F	6.999823	8.243659	6.424256
H	14.972178	10.109378	-0.065104	Cl	13.596992	5.361143	2.551563
H	14.455823	10.950054	1.397645	U	12.405375	6.978540	4.357711

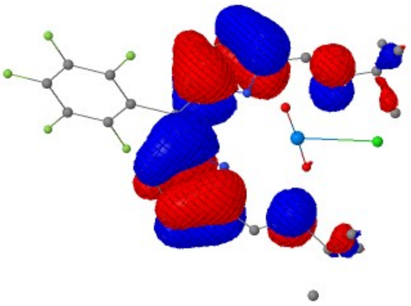

HOMO

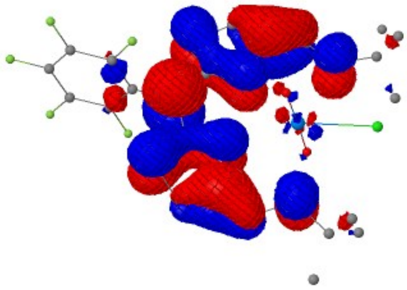

LUMO

3 References

1. G. Sheldrick, *Acta Crystallogr. A*, 2008, **64**, 112.
2. A. Altomare, G. Cascarano, C. Giacovazzo, A. Guagliardi, M. C. Burla, G. Polidori and M. Camalli, *J. Appl. Crystallogr.*, 1994, **27**, 435.
3. L. Farrugia, *J. Appl. Crystallogr.*, 1999, **32**, 837.
4. G. R. Hanson, K. E. Gates, C. J. Noble, M. Griffin, A. Mitchell and S. Benson, *J. Inorg. Biochem.*, 2004, **98**, 903.
5. J. R. Pankhurst, T. Cadenbach, D. Betz, C. Finn and J. B. Love, *Dalton Trans.*, 2015, **44**, 2066.
6. D. M. Barnhart, C. J. Burns, N. N. Sauer and J. G. Watkin, *Inorg. Chem.*, 1995, **34**, 4079.
7. H. Arp, M. Zirngast, C. Marschner, J. Baumgartner, K. Rasmussen, P. Zark and T. Müller, *Organometallics*, 2012, **31**, 4309.
8. M. J. Frisch, G. W. Trucks, H. B. Schlegel, G. E. Scuseria, M. A. Robb, J. R. Cheeseman, G. Scalmani, V. Barone, B. Mennucci, G. A. Petersson, H. Nakatsuji, M. Caricato, X. Li, H. P. Hratchian, A. F. Izmaylov, J. Bloino, G. Zheng, J. L. Sonnenberg, M. Hada, M. Ehara, K. Toyota, R. Fukuda, J. Hasegawa, M. Ishida, T. Nakajima, Y. Honda, O. Kitao, H. Nakai, T. Vreven, J. A. J. Montgomery, J. E. Peralta, F. Ogliaro, M. Bearpark, J. J. Heyd, E. Brothers, K. N. Kudin, V. N. Staroverov, R. Kobayashi, J. Normand, K. Raghavachari, A. Rendell, J. C. Burant, S. S. Iyengar, J. Tomasi, M. Cossi, N. Rega, J. M. Millam, M. Klene, J. E. Knox, J. B. Cross, V. Bakken, C. Adamo, J. Jaramillo, R. Gomperts, R. E. Stratmann, O. Yazyev, A. J. Austin, R. Cammi, C. Pomelli, J. W. Ochterski, R. L. Martin, K. Morokuma, V. G. Zakrzewski, G. A. Voth, P. Salvador, J. J. Dannenberg, S. Dapprich, A. D. Daniels, Ö. Farkas, J. B. Foresman, J. V. Ortiz, J. Cioslowski and D. J. Fox, 2009, Gaussian 09, Revision A.02, Gaussian, Inc., Wallingford CT.
9. (a) A. D. Becke, *J. Chem. Phys.*, 1993, **98**, 5648; (b) J. P. Perdew and Y. Wang, *Phys. Rev. B*, 1992, **45**, 13244.
10. (a) W. Küchle, M. Dolg, H. Stoll and H. Preuss, *J. Chem. Phys.*, 1994, **100**, 7535; (b) X. Cao and M. Dolg, *J. Mol. Struct. (Theochem.)*, 2004, **673**, 203; (c) X. Cao, M. Dolg and H. Stoll, *J. Chem. Phys.*, 2003, **118**, 487.
11. (a) A. Moritz and M. Dolg, *Theor. Chem. Acc.*, 2008, **121**, 297; (b) A. Moritz, X. Cao and M. Dolg, *Theor. Chem. Acc.*, 2007, **118**, 845.

12. (a) L. Castro, A. Yahia and L. Maron, *ChemPhysChem*, 2010, **11**, 990; (b) L. Castro, A. Yahia and L. Maron, *Dalton Trans.*, 2010, **39**, 6682; (c) B. Kosog, C. E. Kefalidis, F. W. Heinemann, L. Maron and K. Meyer, *J. Am. Chem. Soc.*, 2012, **134**, 12792; (d) V. Mougel, C. Camp, J. Pécaut, C. Copéret, L. Maron, C. E. Kefalidis and M. Mazzanti, *Angew. Chem.*, 2012, **124**, 12446; (e) O. Cooper, C. Camp, J. Pécaut, C. E. Kefalidis, L. Maron, S. Gambarelli and M. Mazzanti, *J. Am. Chem. Soc.*, 2014, **136**, 6716.

13. M. Dolg, U. Wedig, H. Stoll and H. Preuss, *J. Chem. Phys.*, 1987, **86**, 866.

14. (a) W. J. Hehre, R. Ditchfield and J. A. Pople, *J. Chem. Phys.*, 1972, **56**, 2257; (b) P. C. Hariharan and J. A. Pople, *Theor. Chim. Acta*, 1973, **28**, 213.
